# Non‐Invasive Auricular Vagus Nerve Stimulation Decreases Heart Rate Variability Independent of Caloric Load

**DOI:** 10.1111/psyp.70017

**Published:** 2025-02-25

**Authors:** Kristin Kaduk, Alessandro Petrella, Sophie J. Müller, Julian Koenig, Nils B. Kroemer

**Affiliations:** ^1^ Department of Psychiatry and Psychotherapy, Tübingen Center for Mental Health University of Tübingen Tübingen Germany; ^2^ German Center for Mental Health (DZPG), partner site Tübingen Tübingen Germany; ^3^ Department of Child and Adolescent Psychiatry, Psychosomatics and Psychotherapy, Faculty of Medicine and University Hospital Cologne University of Cologne Cologne Germany; ^4^ Section of Medical Psychology, Department of Psychiatry and Psychotherapy, Faculty of Medicine University of Bonn Bonn Germany

**Keywords:** autonomic nervous system, cardiac vagal activity, ECG, milkshake, taVNS

## Abstract

The vagus nerve is crucial in regulating physiological functions, including the cardiovascular system. While heart rate (HR) and its variability (HRV) may provide non‐invasive proxies of cardiac vagal activity, transcutaneous auricular vagus nerve stimulation (taVNS) has yielded mixed effects, with limited research on right branch stimulation. In a randomized crossover study with 36 healthy participants, we investigated taVNS effects on HR and HRV indexed by SDRR, RMSSD, HF‐HRV, and LF/HF ratio. To assess the impact of the stimulation side (left vs. right ear) on cardiovascular indices and interaction with the physiological state, we recorded electrocardiograms in four sessions per person, covering three session phases: baseline, during stimulation (taVNS vs. sham), and post‐milkshake consumption with stimulation. First, we found moderate evidence against taVNS affecting HR (BF_10_ = 0.21). Second, taVNS decreased HRV (multivariate *p* = 0.004) independent of physiological state, with strong evidence for RMSSD (BF_10_ = 15.11) and HF‐HRV (BF_10_ = 11.80). Third, taVNS‐induced changes were comparable across sides and stronger than sham, indicating consistent cardiovascular effects independent of the stimulation side. We conclude that taVNS reduces HRV as indexed by RMSSD, HF‐HRV, and SDRR without altering HR, contradicting the assumption that taVNS per se increases cardiovagal activity as indexed by increased HRV due to stimulating vagal afferents. Instead, our results support the role of vagal afferent activation in arousal. Crucially, taVNS on both sides can safely modulate the cardiovascular system without increasing the risk of bradycardia or causing adverse events in healthy participants, offering new treatment possibilities.

## Impact statement

Heart rate variability, measured by HF‐HRV and RMSSD, is conventionally used to index cardiac vagal activity. Our study demonstrates that transcutaneous auricular vagus nerve stimulation (taVNS) decreases HRV indexed by SDRR, RMSSD, and HF‐HRV irrespective of the side of stimulation, contradicting a link to heightened cardiac vagal activity due to stimulating vagal afferents. The findings reveal taVNS's potential as a safe method to modulate cardiovascular function on both sides without increasing the risk of bradycardia or causing adverse events in healthy participants.

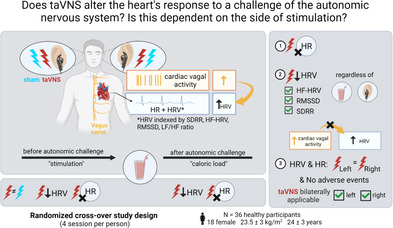

## 1 Introduction

The communication between the brain and the peripheral organs, such as the heart, plays a crucial role in maintaining the body's physiological and metabolic homeostasis (Capilupi et al. 2020). The peripheral organs send interoceptive signals to the brainstem's nucleus of the solitary tract (NTS) through vagal afferents to convey the body's states, such as hunger or alertness (Havel 2001; Maniscalco and Rinaman 2018). Brain signals are then transmitted to peripheral organs via vagal efferents, influencing the gastric frequency (Hong et al. 2019; Teckentrup et al. 2020) or the outflow of the heart by adjusting the firing rate of the pacemaker: the sinus node (Allen et al. 2022; Gourine et al. 2016; Petzschner et al. 2021). Consequently, animal studies have shown that (invasive) stimulation of both right and left cervical vagus nerves affects heart rate (HR) and heart rate variability (HRV), with greater effects often seen on the right side (Huang et al. 2010; Lee et al. 2018; Yoshida et al. 2018).

Despite the vital relevance of brain–body interactions for adaptive human behavior, the impact of vagus nerve stimulation (VNS) on physiological processes and its role in ensuring energy homeostasis is not fully understood (see Burger et al. 2020; Wolf et al. 2021). For example, transcutaneous auricular vagus nerve stimulation (taVNS) allows studying the interaction between the vagus nerve and the heart in humans by non‐invasively stimulating the auricular branch of the vagus nerve in the ear (Butt et al. 2020; Farmer et al. 2021). These acute taVNS studies have yielded mixed results on HRV, with some studies finding increased HRV from stimulating the left side (RMSSD, HF‐HRV & SDRR: Forte et al. 2022; Geng, Liu, et al. 2022) or the right side (RMSSD, SDNN & HF‐HRV: De Couck et al. 2017; Gauthey et al. 2020; RMSSD & SDNN: Machetanz et al. 2021). In contrast, other studies observed a decrease in HRV (RMSSD: Altınkaya et al. 2023; LF/HF ratio: Antonino et al. 2017; Clancy et al. 2014; Weise et al. 2015), whereas most studies found no effect on HRV (Borges et al. 2019; Burger et al. 2019; Šinkovec et al. 2023; Ventura‐Bort and Weymar 2024; Villani et al. 2019) (see Table S1 in the Supporting Information for the taVNS studies with their corresponding HRV indices). This heterogeneity is reflected in a recent meta‐analysis, concluding that taVNS does not robustly alter RMSSD or HF‐HRV in humans (Wolf et al. 2021). While HRV is measured by various cardiovascular indices (e.g., time‐domain measures: SDRR, RMSSD, pNN50; and time‐domain measures: HF‐HRV, LF‐HRV, LF/HF ratio), taVNS might affect these indices differently related to their physiological origin (overview in Box 1). Notably, cardiac vagal modulation through efferent fibers should be explicitly reflected in HF‐HRV, and VNS modulates autonomic nervous system activity by activating afferent vagal fibers. Yet, studies on taVNS and HF‐HRV yield mixed results: Some report an increase in HF‐HRV (Forte et al. 2022; Geng, Liu, et al. 2022), others a decrease in HF‐HRV (Altınkaya et al. 2023), and others found no effect, indicating that the mixed results hold even when considering HF‐HRV as a specific HRV index (see for a comprehensive overview about each HRV index https://neuromadlab.com/en/resources/living‐meta‐analysis‐on‐tvns‐and‐hrv/).

BOX 1 Physiological origin* of several cardiovascular indices (HR and HRV)^1^.

**Table:** 

Heart rate (HR)	Generated by the sinoatrial node, regulated by both branches of the autonomic nervous system^2^ and modulated by circulating hormones, like adrenaline. It is also affected by metabolic and intrinsic factors such as blood pressure and oxygen demand^3^
Root mean of successive differences (RMSSD)	Quantifies the variance in time between successive heartbeats in the time domain and is influenced by cardiac vagal modulation^4^. While RMSSD correlates with HF power, it is less affected by respiration rate^5^
High‐frequency power (HF‐HRV)	Quantifies short‐term periodic dynamics in the heart rate in the frequency domain (high‐frequency power, 0.15–0.40 Hz) and is influenced by cardiac vagal modulation through efferent fibers^6^. The HF‐HRV differs with respiratory rate
Low‐frequency power (LF‐HRV)	Quantifies rhythms with periods between 7 and 25 s in the frequency domain (0.04–0.15 Hz). LF power is modulated by baroreflexes and both cardiac sympathetic and vagal origin^7^
LF/HF ratio	Reflects a mix of sympathetic and parasympathetic activity but is influenced by many other factors. The physiological origin is disputed^8^
Standard deviation of all R‐R intervals (SDRR)	Quantifies all the cyclic components in the heart rate reflecting overall autonomic heart regulation^4,7^ (depends on length of recording period)
^1^Shaffer and Ginsberg (2017); ^2^Armstrong et al. (2022), Boyett (2000); ^3^Green (2011), Kay et al. (1995); ^4^Polanczyk et al. (1998); ^5^Penttilä et al. (2001); ^6^Akselrod et al. (1981), Malik and Camm (1993); ^7^Pomeranz et al. (1985); ^8^Billman (2013)

*Determined by pharmacological blockage studies (gold standard to determine the autonomic origin).

Notwithstanding the inconsistent results across studies, there are open questions concerning the side of stimulation and the body's metabolic state during stimulation. Most taVNS studies have stimulated the left ear, and only one study with right‐sided stimulation and two studies with bilateral stimulation (Bretherton et al. 2019; Clancy et al. 2014; De Couck et al. 2017) could be included in the meta‐analysis (Wolf et al. 2021). Fewer studies use right‐sided taVNS due to a hypothesized higher risk of cardiovascular side effects, such as bradycardia (Kim et al. 2022), and the predominant innervation of the sinus‐atrial node by the right vagus nerve (Ardell and Randall 1986). However, the signal from both auricular branches of the vagus nerve is integrated before activating vagal efferents to the heart, suggesting that side effects may be negligible (Chen et al. 2015; Kim et al. 2022; Redgrave et al. 2018). In addition, small sample sizes (Wolf et al. 2021, meta‐analysis: median (*N*) = 30, range (*N*) = 7–60 of taVNS studies with within‐subject design), lack of a standard stimulation protocol, appropriate baseline measurements, and adequate control conditions may contribute to discrepancies across studies. Likewise, other physiological factors, such as the respiratory rate or hormonal balance (Kozorosky et al. 2022; Sclocco et al. 2019; Szulczewski 2022), interact with the effect of taVNS, indicating a complex interplay between physiological states and taVNS‐induced changes.

The biological behavioral model suggests that after eating, the activity of the vagus nerve helps to regulate energy exchange by coordinating respiratory and cardiovascular processes, ensuring efficient digestion and energy utilization from the consumed food (Grossman and Taylor 2007). Postprandial metabolism is associated with increased heart rate (Ambarish et al. 2005; Chapman et al. 2021; Lu et al. 1999), potentially linked to heightened cardiac sympathetic activity (Nagai et al. 2005; Vaz et al. 1995) to facilitate digestion (Scherrer and Sartori 1997; Van Baak 2008). However, some studies report decreased HF‐HRV (Lu et al. 1999; Ohara et al. 2015) and RMSSD (Chapman et al. 2021) following a caloric load, reflecting reduced cardiac vagal activity. In contrast, Ambarish et al. (2005) observed no significant changes in HR and HRV (SDRR, HF‐HRV, and LF‐HRV) due to food intake, highlighting the complex interplay between metabolism and autonomic responses. Although less is known about the time before caloric intake, one study showed that an increased level of ghrelin, known for regulating metabolism and appetite, decreases heart rate, indicating suppression of cardiac sympathetic nerve activity and increasing SDRR, RMSSD, and HF‐HRV, stimulating cardiac vagal activity (Soeki et al. 2014). Further connected to vagally mediated digestive processes, two studies indicate that increased cardiac vagal activity improves insulin sensitivity, insulin secretion, and glucose tolerance (Heni et al. 2014; Lindmark et al. 2003), which might dominate the later stages of digestion. To sum up, the interplay between metabolism and autonomic responses after a caloric load involves complex shifts between cardiac sympathetic and vagal activity, influenced by factors such as size, composition, and timing of the caloric load. When considering the interaction of metabolism and taVNS, two previous studies reported either non‐significant or inconclusive taVNS‐induced changes in RMSSD after administering a caloric load of ~100 kcal to fasted participants (Altınkaya et al. 2023; Vosseler et al. 2020). Since these studies had a considerably smaller sample size (*N*s = 14 and 15), the interaction with taVNS‐induced changes remains elusive.

To summarize, pressing questions about the differential effects of taVNS at the left vs. the right ear and the emerging evidence of interactions with the metabolic state call for additional studies on autonomic responses to the heart. To close the gap, we employed a randomized crossover design to investigate the effect of stimulation (taVNS vs. sham) on HR and different indices of HRV (RMSSD, SDRR, HF‐HRV, and LF/HF‐ratio) on both auricular branches of the vagus nerve (left vs. right) in different metabolic states, both before and after consuming a milkshake (~400 kcal). We analyzed several cardiovascular indices to capture various aspects of cardiac autonomic regulation (overview about the physiological origin in Box 1): HF‐HRV, closely linked to respiration rate, and RMSSD, both of which reflect short‐term beat‐to‐beat variations (Shaffer and Ginsberg 2017); SDRR, which provides an overall measure of R‐R interval variability over the entire 30 min stimulation period; and the LF/HF ratio, as it has been commonly reported in many taVNS studies, according to the meta‐analysis by Wolf et al. (2021). By combining these measurements with heart rate and respiration rate, we can evaluate the autonomic responses of the heart following VNS in different metabolic states. For example, early stages of digestion may be linked to withdrawal of cardiac vagal activity, inhibiting the heart's pacemaker (Ng et al. 2001) and potentially increasing heart rate after the caloric load. While the interplay between taVNS and metabolism remains unclear, we are using a milkshake load to evaluate taVNS‐induced changes through different phases of autonomic regulation, which may help resolve inconsistent effects of taVNS on HRV across previous studies.

## 2 Methods

We preregistered our study protocol at the Open Science Framework (https://osf.io/26v5n, February 2021). The reported ECG data are part of a larger study examining whether the effects of taVNS on physiological parameters reflecting digestion, food reward, and mood are lateralized, as suggested by animal studies (Anselmi et al. 2017; Brougher et al. 2021; Han et al. 2018).

### 2.1 Participants

The sample size was selected to provide at least a power of 1−*β* = 0.90 for medium‐sized within‐subject effects (Cohen's *f* = 0.15, *dz* ~0.57). We performed an a priori power calculation using G*Power (ver. 3.1.9.6, Faul et al. 2007) and set the following parameters (significance level: *α* = 0.05, statistical test: repeated measures within‐subject effects, number of groups: 2, number of measurements: 4, correlation among repeated measures: 0.8), leading to a lower‐bound estimate of 34 participants after quality control. A total of 38 participants were invited to participate. One participant was excluded due to paraesthesia experienced in the sham condition, and another participant was excluded due to undetectable R‐peaks in two sessions. Thus, 36 participants were included in the final analysis (18 women, *M*_age_ = 24 ± 3 years, BMI: 23.5 ± 2.6 kg/m^2^, 87.2% of the sample were students).

Participants were recruited by the university mailing list of the University of Tübingen, social media, and flyers distributed in public institutions. Participants underwent a comprehensive screening process to ensure eligibility for the study. According to the screenings, all participants were physically healthy and excluded if they had contraindications for taVNS (requiring permanent use of hearing aids, having open wounds or impaired skin at the electrode side, irremovable earrings or piercings on the ear and implants such as a pacemaker, cochlear implant, cerebral shunt, pregnant, or nursing). This was followed by a psychological assessment using the Structured Clinical Interview for DSM (SCID) to exclude any potential psychiatric disorder. Additional exclusion criteria included the acute presence of metabolic and digestive disorders, acute intake of medication that potentially interfered with the electrogastrogram (prokinetics, anti‐inflammatory agents, i.e., NSARs, etc.), lifetime presence of asthma, brain injury, cardiovascular diseases, occurred apoplexy, schizophrenia, bipolar disorder, as well as 12‐month prevalence of eating disorder, somatic symptom disorder, obsessive‐compulsive disorder, and severe substance use disorder. After completing the fourth session, participants received compensation of 80€ or partial course credits of 4 h and 40€. All participants provided their written informed consent before the experiment. The ethics committee of the Faculty of Medicine at the University of Tübingen approved the experiment, and all procedures were carried out in accordance with the Declaration of Helsinki.

### 2.2 Procedure

**FIGURE 1:**
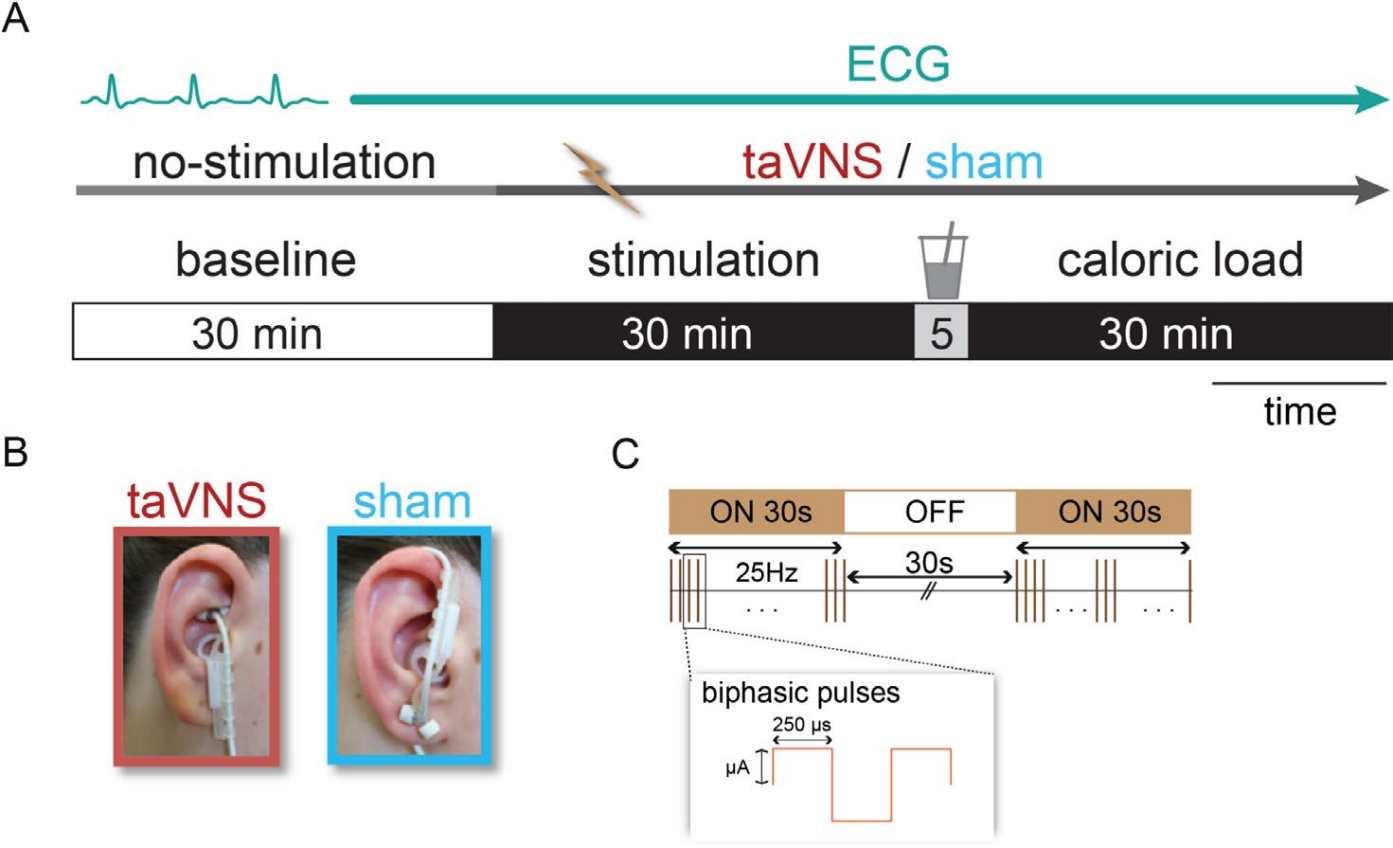
Schematic summary of the experiment. (A) The timeline of a session includes the phases of baseline (30 min, no stimulation) followed by stimulation (30 min) when either taVNS or sham was applied. Participants received a high‐caloric load to challenge the autonomic system, while we collected the phase after the caloric load (30 min) with concurrent stimulation. (B) The electrode locations at the cymba conchae for the transcutaneous auricular vagus nerve stimulation (taVNS, red) and the ear lobe for sham stimulation (blue) are displayed. (C) Illustration of the stimulation protocol with a biphasic impulse frequency of 25 Hz, the pulse width of 250 μs with alternating intervals of 30 s stimulation on and 30 s stimulation off. The stimulation intensity (mA) was adjusted for each participant until they felt a “mild pricking”.

To maximize power and account for inter‐individual variability, we adopted a randomized crossover design. The three within‐subject factors were stimulation side (right, left), stimulation (taVNS, sham), and time (baseline, stimulation, caloric load), leading to four sessions per participant. Each session had the same timeline (Figure 1A), starting with a no‐stimulation baseline phase while recording peripheral physiological signals (i.e., electrocardiogram (ECG) and electrogastrogram (EGG)) for 30 min. Participants answered questions on positive and negative affective states (PANAS, Watson et al. 1988), food cravings (FCQ‐Tr, Meule et al. 2014), and metabolic parameters (i.e., hunger, thirst, and satiety) every 15 min. At the end of each session, participants completed two tasks (willingness‐to‐pay for food (Plassmann et al. 2007) and body silhouette task (Nummenmaa et al. 2014)), as detailed in our preregistered study protocol.

To collect ECG, we continuously measured the heart's electrical activity at 5000 Hz using three bipolar electrodes connected to a BrainAmp amplifier (Brain Products, Germany). Electrodes that shared the same negative derivation were placed on the left and right sides of the clavicle pits. The positive derivations were set on the left side in the seventh intercostal space, as previously described by Teckentrup et al. (2020). ECG recordings continued while participants received taVNS or sham stimulation for 30 min before and after administering a standardized caloric load (~400 kcal milkshake, details in Supporting Information). Participants were instructed to attend the session 2–3 h after their last meal. To account for circadian rhythms, we scheduled the four experimental sessions for each participant at roughly the same time across different days (*M*_del_t_ = 23.98 min (range: 0–104 min)). The majority of first sessions took place in the afternoon (2 to 6 pm, *n* = 19), followed by noon (10 am to 2 pm, *n* = 12), morning (6 to 10 am, *n* = 9), and only one session started after 6 pm. Except for state questions every 15 min (when the neutral‐toned soundtrack “Leaving Hogwarts” was played), participants rested and listened to the audiobook “Harry Potter and the Philosopher's Stone”, a fantasy novel by J.K. Rowling, chosen for its engaging yet non‐arousing content.

### 2.3 Electrical Stimulation

To stimulate the auricular branch of the vagus nerve, we used the NEMOS device and the NEMOS electrode (cerbomed GmbH, Erlangen, Germany) following the procedure of previous studies (Ferstl et al. 2021; Frangos et al. 2015; Neuser et al. 2020; Teckentrup et al. 2021). For taVNS, the electrode was placed at the cymba conchae (Peuker and Filler 2002) as it induces robust activation in the NTS (Borgmann et al. 2021; Frangos et al. 2015; Teckentrup et al. 2021; Yakunina et al. 2017). The electrode was turned upside down for sham stimulation and placed at the earlobe (Butt et al. 2020) (Figure 1B). Subjects with extensive body hair were instructed in advance to shave to enable better retention of the electrodes. In addition, the skin below the electrodes was wiped with a disinfecting wipe to remove residual lipids for better skin conductivity, and we applied electrode contract spray (vyaire, Helsinki, Finland) on the electrode pulsers, which cover the two titan parts of the electrode without creating an electric circuit bridge.

Analogous to previous studies, the intensity increased from 0.1 in 0.1 mA increments until participants reported a “pricking” sensation that was not painful. Each participant's stimulation intensity (Figure 1C) was adjusted to a “mild pricking” level before the onset of the experiment, corresponding to a mid‐point value on the visual analog scale (VAS) ranging from no sensation (0) to light sensation (1), light tingling (2), moderate tingling (3), strong tingling (4), light pricking (5), moderate pricking (6), pricking (7), pain (8), unbearable pain (9), and strongest imaginable sensation (10). The stimulation intensities were matched between taVNS and sham on the perceptual level (Table S2 in the Supporting Information). During the sessions, participants reported no adverse effects of taVNS (e.g., pain, headache, and dizziness).

The side of stimulation was randomized in advance. To ensure that taVNS and sham are comparable on each side, the stimulation side was not changed between Sessions 1 and 2 (i.e., if a participant received left‐sided taVNS in Session 1, they received sham on the left side in Session 2). Participants were blinded regarding the stimulation condition and asked to guess if their stimulation was real or sham after each session. Of 145 total guesses, only 66 were correct, yielding an accuracy of 45.5%, which is not better than chance.

### 2.4 Data Analysis

The analyses of the ECG data aim to close the research gap on the potentially lateralized effects of non‐invasive taVNS on HR and different parameters of HRV. The study was preregistered before we conducted the living Bayesian meta‐analyses of taVNS‐induced changes in RMSSD and HF‐HRV (Wolf et al. 2021), so we did not include the ECG analyses in the preregistration at the time. The ECG data from the current study are openly available as a resource on OSF (https://osf.io/26v5n).

### 2.5 R‐Peak Detection and Cardiovascular Indices

All cardiovascular indices were derived from continuous ECG recordings. The ECG signal was extracted and down‐sampled to 1000 Hz using the FieldTrip toolbox (Oostenveld et al. 2011, http://fieldtriptoolbox.org). Custom code (https://github.com/dagdpz/body_signals_analysis) was partly adjusted and used for the pre‐processing and R‐peak detection. We processed the recorded and detrended ECG data to remove the 50 Hz power line interference (19th order Butterworth filter with a passband of 40 Hz, stop band of 100 Hz, passband ripple of 1 dB, and stopband ripple of 150 dB) and baseline drifts (high‐pass filter with a cut‐off frequency of 0.5 Hz), which can affect the accuracy of peak detection. We then detected the R‐peaks and QRS complex and computed the R‐R interval time series. As the ECG morphology is strongly affected by movement artifacts, we used an automatic procedure to check the R‐peaks and the R‐R intervals for robustness and deviations (see Supporting Information). All detected deviations and their consecutive R‐R intervals were deleted from the signal. On average, 97.1% (1769s, range: 81%–100%) of a 30‐min block of ECG recordings was further analyzed to derive cardiovascular indices (except for one phase with 956 s of ECG data).

HRV is the variation in time between consecutive heartbeats. Four standard HRV measures are, according to our meta‐analyis, commonly reported in taVNS studies: the SDRR, RMSSD, HF‐HRV, and LF/HF ratio (Wolf et al. 2021). We computed the following time‐domain measures over the entire stimulation period per condition: the standard deviation of R‐R intervals (SDRR in ms) was calculated to capture slow fluctuations as a general trend over the entire stimulation period per condition (around 30 min). Because of the SDRR's dependency on the length of the recording period, particularly when the total variance of HRV increases with the duration of analysis, it is difficult to compare between studies. The root mean squares of successive differences of adjacent heartbeats (RMSSD in ms) represent the beat‐to‐beat variance in the heart period. For frequency‐domain HRV, we computed the spectra using the HRV toolbox of the PhysioNet Cardiovascular Signal Toolbox (Vest et al. 2018) in 6 min windows of 30 s steps, after cubic spline interpolation to account for potential movement artifacts during questionnaires, using Welch's method. For each time window, 20% of data could be rejected before a window is considered too low quality for analysis. To ensure that the length of the window does not drop below 5 min of data, given that 20% of the signal can be rejected before a window is considered low quality for analyses, we choose a maximum 6 min window for analyses. Two frequency‐domain HRV measurements were computed from R‐R intervals based on power spectral density: High‐frequency HRV (HF‐HRV) at 0.15–0.4 Hz and low‐frequency HRV (LF‐HRV) at 0.04–0.15 Hz, following recommendations from the Task Force of the European Society of Cardiology and the North American Society of Pacing and Electrophysiology. For completeness, we added the main statistical results of the LF‐HRV as Table S5 to the Supporting Information.

### 2.6 Extraction of Respiration Signal from the R‐R Intervals

To evaluate taVNS‐induced changes in the respiration rate, we employed the Neurophysiological Signal Processing toolbox (NeuroKit2, Makowski et al. 2021) to extract the respiration signal from the ECG. Respiratory rates were estimated from the variations in R‐R intervals using the default method (Van Gent et al. 2019) to extract the respiratory rate over periods of 6 min in steps of 30 s for each phase (baseline, stimulation, caloric load).

### 2.7 Statistical Analysis

The statistical analysis was performed using R (version 4.1.2, R Core Team, 2022). Cardiovascular indices (HR, HRV) were baseline‐corrected by subtracting the individual session‐specific baseline index from later indices (separately for stimulation and caloric load phases). However, the raw data are displayed in the Supporting Information (see Figure S1, see Table S3 for the mean and standard deviation for the session‐specific baseline, and see Table S4 for the results of the MANOVA for the baseline). Additionally, we also present the average changes in cardiovascular indices for both taVNS and sham conditions at the onset of stimulation over time (see Figure S2).

For the analysis of the HR (preregistered as a secondary outcome), we investigated the effect of the three within‐subject factors (stimulation (taVNS, sham) × side (right, left) × phase (stimulation, caloric load)) using bootstrapping. For the analyses of the HRV, we computed four HRV indices (SDRR, RMSSD, HF‐HRV, and LF/HF ratio), which were the most common measures of HRV according to our meta‐analysis (Wolf et al. 2021). To assess the impact of the three within‐subject factors (stimulation (taVNS, sham) × side (right, left) × phase (stimulation, caloric load)) on the four dependent HRV indices, we conducted a multivariate analysis of variance (MANOVA) first. Next, we investigated the effects on each HRV index separately using bootstrapping. To prepare the bootstrapping analyses, we calculated the net effect of stimulation by subtracting baseline‐corrected sham from baseline‐corrected taVNS (pairwise differences) for each participant and session. To avoid distributional assumptions, we bootstrapped parameter distribution for statistics (e.g., Teckentrup et al. 2020, 50,000 resampling steps). The result section starts with the main effects, where individual estimates were averaged first to test the effects across stimulation and sides. Analogously, we tested potential associations with respiration rate and the covariates sex, BMI, and age as post hoc control analyses. As a threshold, we used *p* ≤ 0.05 (two‐tailed). To address dependencies in multiple comparisons, we applied the Benjamini & Hochberg method to adjust p‐values for the post hoc tests. To quantify the effect of taVNS on HR and HRV, we calculated effect sizes (Cohen's *dz*) and carried out Bayesian one‐sample *t*‐tests, including individual estimates of taVNS‐induced changes in each HRV index per stimulation side (left and right). To reflect that small‐to‐moderate effects are likely, we used a Cauchy prior with a width of 0.5 in JASP v.0.17.2 (Quintana and Williams 2018). The Bayesian factors were interpreted according to the reference criteria from Harold Jeffreys (Jeffreys 1939).

## 3 Results

### 3.1 Stimulation Decreases HR, While Caloric Load Increases It

**FIGURE 2:**
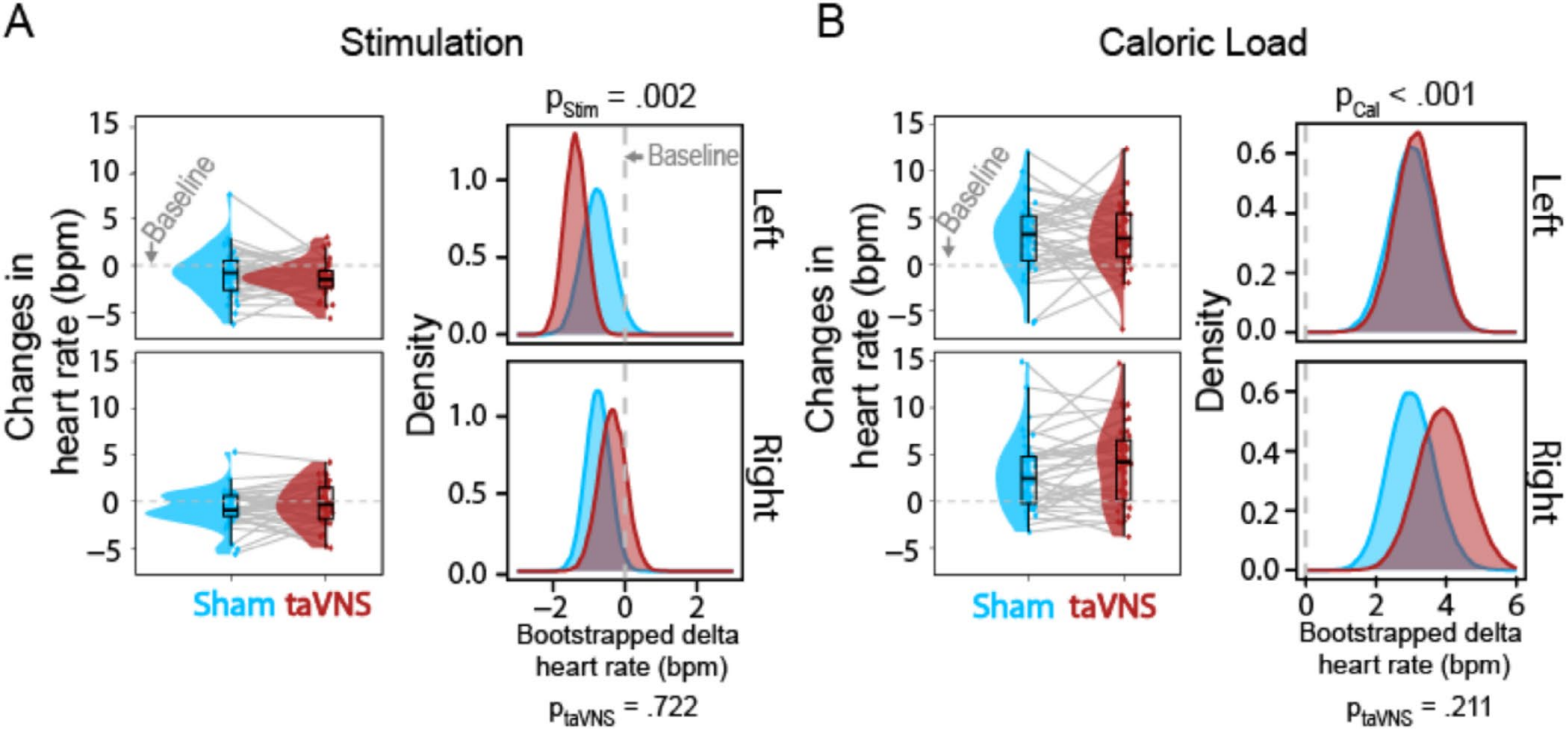
Stimulation decreases and caloric load increases heart rate (HR). (A) During stimulation, HR decreases across both sides (left vs. right) and both stimulation conditions (taVNS in red vs. sham in blue), indicated by the *p*‐values below the heading. We observed no significant differences between stimulation conditions on each side, indicated by the bootstrapped distributions of changes relative to baseline. (B) After the caloric load, HR increases across both sides (left and right) and stimulation conditions (taVNS and sham), as indicated by the *p*‐values below the heading. We observed no significant differences between stimulation conditions on each side, indicated by the bootstrapped distributions of changes relative to baseline. The dots depict each participant's change in average HR for taVNS (in red) and sham (in blue) compared to the baseline.

To evaluate the effect of stimulation (taVNS vs. sham; left and right side) before administering the caloric load, we analyzed changes in HR during the 30 min stimulation phase versus baseline. Across both stimulation conditions, HR decreased during stimulation (Figure 2A; *b* = −0.82 bpm, 95% CI [−1.35, −0.30], *p*_Boot_ = 0.002, *p*_H&B_ = 0.004). However, before the challenge, we did not observe significant differences between taVNS and sham across both sides (*b* = −0.11 bpm, 95% CI [−0.70, 0.46], *p*_Boot_ = 0.722, *p*_H&B_ = 0.722).

Next, to evaluate the interaction with a challenge of the autonomic system, we analyzed the main effect of caloric load on HR. As expected, HR increased after consuming a 400‐kcal milkshake (Figure 2B; *b* = 3.28 bpm, 95% CI [2.33, 4.21], *p*_Boot_ < 0.001, *p*_H&B_ < 0.001; relative to the stimulation phase: *b* = 4.09 bpm, 95% CI [3.36, 4.85], *p*_Boot_ < 0.001, *p*_H&B_ < 0.001). We also did not observe significant differences between taVNS and sham across both sides (*b* = 0.49 bpm, 95% CI [−0.28, 1.25], *p*_Boot_ = 0.211, *p*_H&B_ = 0.602). These results suggest that stimulation and caloric load altered HR within a session, but these changes were not specific to taVNS, as suggested by the moderate evidence against an effect (Bayes factor, BF_10_ = 0.21).

### 3.2 Stimulation and Caloric Load Modulate HRV

Since the different HRV indices (SDRR, RMSSD, HF‐HRV, and LF/HF ratio) are correlated measures of a similar construct, we initially ran a multivariate analysis to evaluate taVNS‐induced changes among all HRV indices. HRV was modulated by both the stimulation (MANOVA, *V*_Pillai's Trace_ = 0.053, *F*(4,276) = 3.88, *p* = 0.004) and caloric load (*V*_Pillai's Trace_ = 0.215, *F*(4,277) = 18.97, *p* < 0.001), but the side of stimulation did not contribute to the changes in HRV, and there was no interaction of stimulation with metabolic state (*p*s > 0.05).

### 3.3 taVNS Reduces SDRR, RMSSD, and HF‐HRV Before the Caloric Load

**FIGURE 3:**
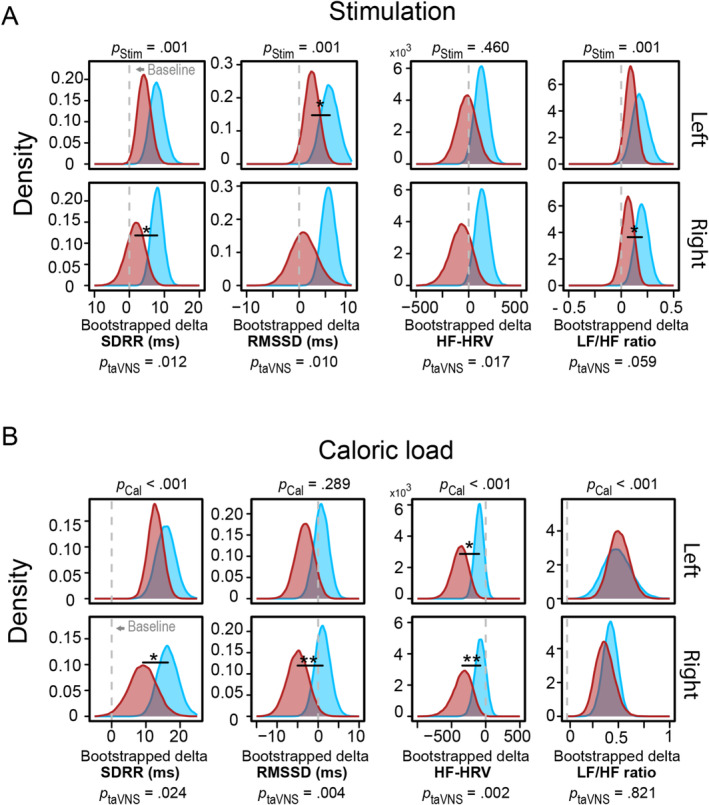
taVNS decreases HRV as indexed by SDRR, RMSSD, and HF‐HRV. (A) Across both stimulation conditions (taVNS vs. sham), SDRR, RMSSD, and LF/HF ratio are altered across both sides (left vs. right) before the caloric load, indicated by the *p*‐values above each plot. taVNS (red distribution) decreased SDRR, RMSSD, and LF/HF ratio side‐specific compared to sham (blue distribution) before the challenge, indicated by the bootstrapped distributions of the delta to the baseline separated for each side. Statistics for the taVNS‐induced effects across both sides are displayed below the panel. (B) The caloric load alters SDRR, HF‐HRV, and LF/HF ratio, except for RMSSD, across both sides (left vs. right) and stimulation conditions (taVNS vs. sham), indicated by the *p*‐values above each plot. taVNS decreased SDRR, RMSSD, and HF‐HRV after the caloric load compared sham, as indicated by the bootstrapped distributions.

To complement the multivariate findings and to compare taVNS‐induced effects on HRV with prior studies, we computed four commonly reported HRV measures used in most taVNS studies (Wolf et al. 2021). We analyzed changes in SDRR, RMSSD, HF‐HRV, and RMSSD during the 30‐min stimulation phase versus baseline (i.e., analogous to post hoc tests). Before the caloric load, stimulation (i.e., taVNS and sham) increased SDRR (Figure 3A; *b* = 5.47 ms, 95% CI [2.90, 8.22], *p*_Boot_ < 0.001, *p*_H&B_ < 0.001), RMSSD (*b* = 3.68 ms, 95% CI [1.40, 6.20], *p*_Boot_ = 0.001, *p*_H&B_ = 0.002) and LF/HF ratio (*b* = 0.13, 95% CI [0.05, 0.22], *p*_Boot_ = 0.001, *p*_H&B_ = 0.002), but not HF‐HRV (*b* = 38.83 ms^2^, 95% CI [−63.21, 140.89], *p*_Boot_ = 0.460, *p*_H&B_ = 0.460).

Next, we investigated the effect of taVNS on the individual HRV indices (SDRR, RMSSD, HF‐HRV, and RMSSD) by phase. Notably, in the time domain, we observed a taVNS‐induced decrease in SDRR (Figure 3A; *b* = −4.98 ms, 95% CI [9.56, −1.04], *p*_Boot_ = 0.012, *p*_H&B_ = 0.034) and RMSSD across both sides (*b* = −4.05 ms, 95% CI [−7.44, −0.92], *p*_Boot_ = 0.010, *p*_H&B_ = 0.034). In the frequency domain, taVNS reduced HF‐HRV across sides (*b* = −174.84 ms^2^, 95% CI [−365.75, −24.65], *p*_Boot_ = 0.017, *p*_H&B_ = 0.034), which was more pronounced at the right ear (Table 1). To summarize, SDRR, RMSSD, and HF‐HRV decreased during taVNS compared to sham and there was no interaction with the stimulation side.

**TABLE 1:** Comparison taVNS versus sham during the stimulation phase after bootstrapping.

Index	Both sides			Left		Right	
	Mean [CI_L_, CI_U_]	*p*	Effect size *dz*	Mean [CI_L_, CI_U_]	*p*	Mean [CI_L_, CI_U_]	*p*
Stimulation							
HR	−0.11 [−0.70, 0.46]	0.722	0.058	−0.61 [−1.40,0.18]	0.128	0.40 [−0.25, 1.17]	0.305
SDRR	**−4.98 [−9.56, −1.04]**	**0.012**	**0.471**	−3.69 [−8.03, 0.60]	0.092	**−6.28 [−12.84, −0.78]**	**0.022**
RMSSD	**−4.05 [−7.44, −0.92]**	**0.010**	**0.441**	**−3.39 [−6.54, −0.56] −0.51]**	**0.017**	−4.70 [−10.32, 0.64]	0.088
HF‐HRV	**−174.84 [−365.75, −24.65]**	**0.017**	**0.415**	−143.75 [−353.64, 45.97]	0.147	−206.04 [−448.15, 2.88]	0.054
LF/HF ratio	−0.11 [−0.25, 0.00]	0.059	0.347	−0.09 [−0.27, 0.08]	0.300	**−0.14 [−0.29, −0.00]**	**0.048**
Respiration	0.03 [−0.59, 0.66]	0.913	−0.026	−0.22 [−0.74, 0.23]	0.375	0.26 [−0.15, 0.67]	0.211
Caloric load							
HR	0.49 [−0.28, 1.25]	0.211	−0.155	0.10 [−1.26, 1.42]	0.882	0.89 [−0.24, 1.98]	0.123
SDRR	**−5.16 [−10.07, −0.63]**	**0.024**	**0.318**	−3.02 [−7.48, 1.44]	0.184	**−7.25 [−14.91, −0.51]**	**0.034**
RMSSD	**−5.09 [−8.83, −1.54]**	**0.004**	**0.460**	−4.07 [−9.02, 0.76]	0.101	**−6.13 [−11.02, −1.63] −1.74]**	**0.006**
HF‐HRV	**−271.54 [−477.26, −95.60]**	**0.002**	**0.447**	**−279.61 [−550.76, −30.18]**	**0.027**	**−264.05 [−475.41, −75.68]**	**0.005**
LF/HF ratio	−0.02 [−0.16, 0.13]	0.821	0.032	0.02 [−0.19, 0.23]	0.845	−0.05 [−0.20, 0.10]	0.492
Respiration	−0.21 [−1.17, 0.84]	0.660	0.076	−0.20 [−0.90, 0.50]	0.566	−0.01 [−0.61, 0.62]	0.974

*Note:* CI_L_ = 95% lower bound and CI_U_ = 95% upper bound of confidence interval. Bold values indicate significant differences (*p* < 0.05).

### 3.4 taVNS Robustly Affected SDRR, RMSSD, and HF‐HRV After a Caloric Load

To investigate HRV indices after an autonomic challenge, we first evaluated the effect of a standardized caloric load on SDRR, RMSSD, HF‐HRV, and LF/HF ratio. After the challenge, we observed a decrease in HF‐HRV (Figure 3B; *b* = −223.29 ms^2^, 95% CI [−378.31, −92.20], *p*_Boot_ < 0.001, *p*_H&B_ < 0.001) and an increase in SDRR (*b* = 13.56 ms, 95% CI [9.02, 18.29], *p*_Boot_ < 0.001, *p*_H&B_ < 0.001) and LF/HF ratio (*b* = 0.45, 95% CI [0.31, 0.60], *p*_Boot_ < 0.001, *p*_H&B_ < 0.001) compared to baseline. In contrast, the challenge did not affect RMSSD (*b* = −1.58 ms, 95% CI [−4.63, 1.26], *p*_Boot_ = 0.289, *p*_H&B_ = 0.289).

Regarding the time‐domain measures of HRV, taVNS reduced SDRR (Figure 3B, *b* = −5.16 ms, 95% CI [−10.07, −0.63], *p*_Boot_ = 0.024, *p*_H&B_ = 0.048) and RMSSD across both sides (*b* = −5.09 ms, [−8.83, −1.54], *p*_Boot_ = 0.004, *p*_H&B_ = 0.012) after the caloric load. Regarding the frequency‐domain measures of HRV, taVNS reduced HF‐HRV after the caloric load across both sides (*b* = −271.54 ms^2^, [−477.26, −95.60], *p*_Boot_ = 0.002, *p*_H&B_ = 0.012) and for each side (Table 1). However, this did not lead to taVNS‐induced changes in the LF/HF ratio across both sides (*b* = −0.02, [−0.16, 0.13], *p*_Boot_ = 0.821, *p*_H&B_ = 0.821). To summarize, taVNS at both ears reduced SDRR, RMSSD, and HF‐HRV after the caloric load, replicating the effects in independent sessions within the same sample.

To investigate the potential variance in taVNS effects on all HRV indices across different physiological conditions, we compared each HRV index between the stimulation and caloric load phases. RMSSD and HF‐HRV decreased after the caloric load compared to the stimulation (RMSSD: *b* = −5.27 ms, 95% CI [−8.00, −2.59], *p*_Boot_ < 0.001, *p*_H&B_ = 0.001; HF‐HRV: *b* = −262.23 ms^2^, 95% CI [−374.95, −154.94], *p*_Boot_ < 0.001, *p*_H&B_ = 0.001) and SDRR and LF/HF ratio increased (SDRR: *b* = 8.10 ms, 95% CI [3.65, 12.77], *p*_Boot_ < 0.001, *p*_H&B_ = 0.001; LF/HF ratio: *b* = 0.31, 95% CI [0.21, 0.42], *p*_Boot_ < 0.001, *p*_H&B_ = 0.001). After the caloric load, we found that taVNS decreased HF‐HRV and RMSSD, further amplifying the reduction in RMSSD and HF‐HRV induced by the milkshake. Notably, the taVNS‐induced effects in the caloric load phase did not significantly differ from the taVNS‐induced effects in the stimulation phase for any HRV index (*p*_Boot_ > 0.05, see Table S8 in the Supporting Information). To summarize, taVNS consistently decreased SDRR, RMSSD, and HF‐HRV, irrespective of the physiological state, as shown by strong evidence, particularly for RMSSD (BF_10_ = 15.11) and HF‐HRV (BF_10_ = 11.80).

### 3.5 Respiration Rate Decreases After Stimulation, But No Link to taVNS‐Induced Changes in HF‐HRV

As HF‐HRV and respiration are closely linked, we extracted the respiration rate from the R‐R intervals to evaluate the stimulation effects on respiration rate and the modulatory effect of respiration on HF‐HRV. Across both stimulation conditions, respiration rate decreased during stimulation (*b* = −0.23 cpm, 95% CI [−0.38, −0.09], *p*_Boot_ = 0.006, *p*_H&B_ = 0.008), but not after the caloric load (*b* = −0.29 cpm, 95% CI [−0.67, 0.07], *p*_Boot_ = 0.112, *p*_H&B_ = 0.134). We did not observe significant differences in the respiration rate between taVNS and sham across both sides before the caloric load (*b* = −0.03 cpm, 95% CI [−0.59, 0.66], *p*_Boot_ = 0.913, *p*_H&B_ = 0.913), nor after the caloric load (*b* = −0.21 cpm, 95% CI [−1.17, 0.84], *p*_Boot_ = 0.660, *p*_H&B_ = 0.792). To evaluate the relationship between HF‐HRV and respiration rate, we correlated the taVNS/sham‐induced changes in respiration rate and HF‐HRV. We found no significant relationship between taVNS/sham‐induced changes in respiration rate and HF‐HRV (*p* > 0.05), indicating that any variation in respiratory rate across conditions did not confound our main outcome about the effects of taVNS on HF‐HRV, RMSSD, and SDRR. In addition, baseline indices of HR and HRV were also not associated with BMI, age, or sex (*p* > 0.05).

### 3.6 No Side‐Specific Differences in HR and HRV Indexed by SDRR, RMSSD, HF‐HRV, and LF/HF Ratio Due to taVNS

**FIGURE 4:**
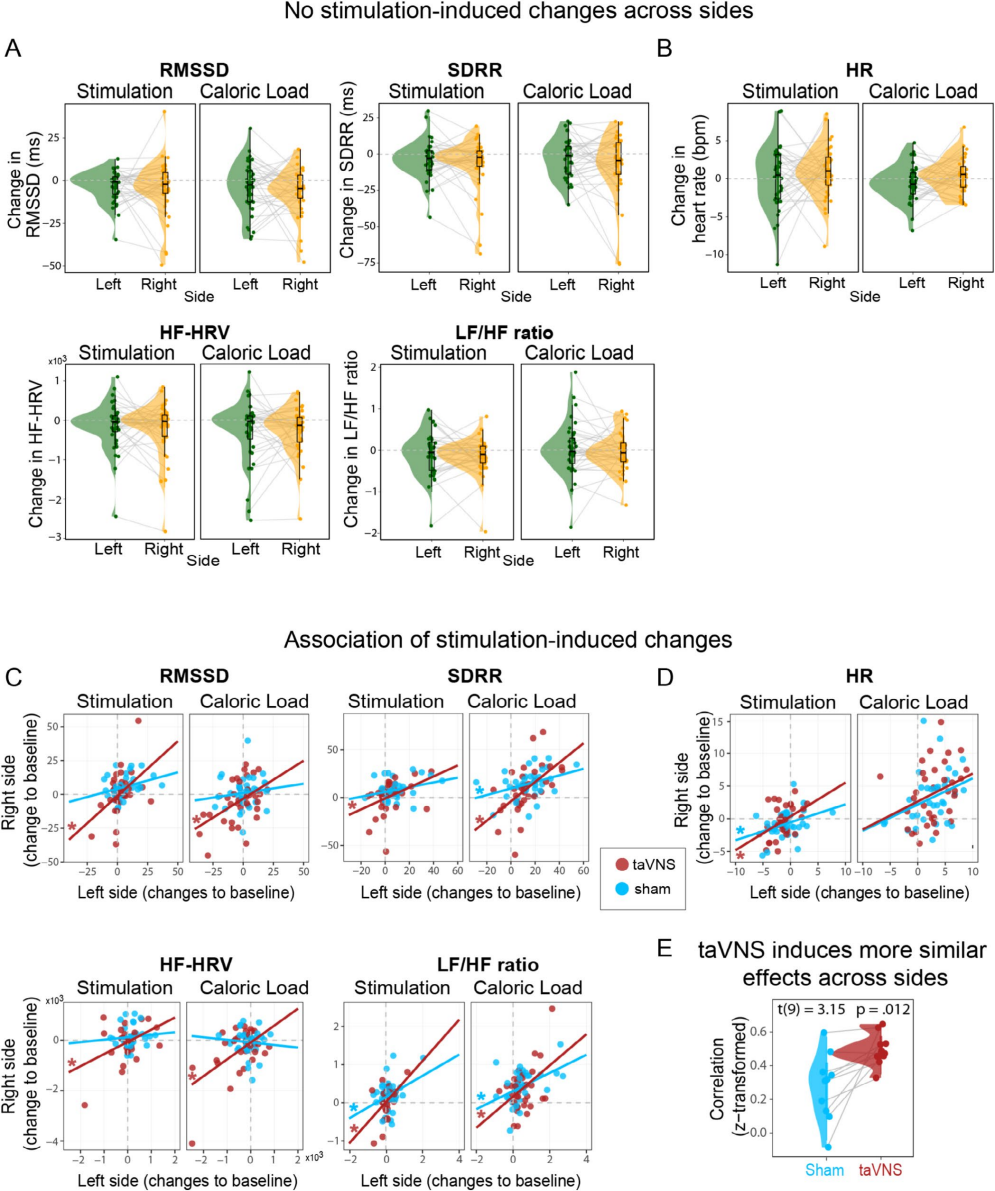
No differences between the sides of vagus nerve stimulation in heart rate and heart rate variability (SDRR, RMSSD, HF‐HRV, LF/HF ratio). (A, B) For cardiovascular indices, dots depict the participant's average score for SDRR, RMSSD, HF‐HRV, LF/HF ratio, and HR (taVNS—sham of their change from baseline), and the group distribution shows no significant difference for the side‐specific comparison (left vs. right) for either the stimulation or caloric load phases. (C, D) TaVNS‐induced changes (red) in SDRR, RMSSD, HF‐HRV, LF/HF ratio, and HR are more strongly correlated across sides compared to sham (blue), indicated by the stars at the correlation lines for the data of each participant. (E) Higher correlation coefficients for cardiovascular indices between sides of stimulation after taVNS compared to sham.

To better understand the lateralization effects of taVNS, we investigated whether left or right taVNS affects HR or HRV (SDRR, RMSSD, HF‐HRV, and LF/HF ratio) differently. In contrast to the theorized larger risk of right taVNS for cardiac function, there was no significant difference in any HRV index (Figure 4A; *p*s_Boot_ > 0.05) or HR (Figure 4B; *p*_Boot_ > 0.05), indicating similar cardiovascular effects across both sides (anecdotal evidence against an effect, Bayes factor, BFs_10_ < 0.6, see Table S7 in the Supporting Information). For 9 out of 10 computed cardiovascular indices, the changes in SDRR, RMSSD, HF‐HRV, and LF/HF ratio (Figure 4C; see Table S6 in the Supporting Information), and HR (Figure 4D; see Table S6) correlated more strongly across sides for taVNS versus sham (one‐sample *t*‐test of the Fisher *z*‐transformed coefficients, *t*(9) = 3.16, *p* = 0.012, Figure 4E).

## 4 Discussion

The cardiovascular system is regulated by bidirectional communication via the vagus nerve with the brain. Although HF‐HRV and RMSSD are established measures of cardiac vagal activity, as demonstrated in multiple pharmacological blockage studies (Laborde et al. 2017; Pomeranz et al. 1985), previous studies investigating the effect of stimulating vagal afferents using taVNS on HF‐HRV and RMSSD in humans have yielded inconclusive results. To address this gap, we used a single‐blind randomized crossover design to evaluate the side‐ and state‐specific effects of taVNS on changes in HR and HRV indexed by SDRR, RMSSD, HF‐HRV, and LF/HF ratio (i.e., within participants). Although the stimulation and the caloric load as experimental manipulations altered HR, we found no effect of taVNS (vs. sham). Contrary to theorized heightened cardiac vagal activity due to taVNS, stimulating vagal afferents did not increase HRV. Instead, taVNS decreased HRV as indexed by SDRR, RMSSD, and HF‐HRV before and after the caloric load, indicating a successful modulation of cardiac function in both states. Intriguingly, we observed comparable taVNS‐induced changes across sides that were more strongly correlated than sham‐induced changes. This supports the interpretation that side‐specific vagal afferent inputs through the auricular branch are integrated into the brain for cardiovascular control before relaying them efferently to the body. We conclude that taVNS‐induced changes in HR and HRV indexed by SDRR, RMSSD, and HF‐HRV are largely independent of the side of the stimulation and the body's metabolic state. Crucially, our results may help reconcile acute modulatory effects of the cardiovascular system facilitating arousal, not relaxation, with VNS‐induced increases in pupil dilation (D'Agostini et al. 2023; Lloyd et al. 2023; Sharon et al. 2021; Skora et al. 2024), invigoration (Neuser et al. 2020), and VNS‐induced decreases in alpha oscillations in an EEG (Chen et al. 2023; Lewine et al. 2019; Sharon et al. 2021, in contrast to no significant effect by Lloyd et al. 2023) that are not well explained by parasympathetic activation of a “rest and digest” mode (Teckentrup and Kroemer 2024).

Consistent with recent findings, we observed a robust taVNS‐induced reduction in SDRR, HF‐HRV, and RMSSD across both sides (Altınkaya et al. 2023; Clancy et al. 2014; Weise et al. 2015). These findings contradict the assumption that stimulating vagal afferents leads to higher HF‐HRV, typically interpreted as indicating sustained cardiac vagal activity (Shaffer and Ginsberg 2017). This relationship was previously demonstrated by increased compound action potentials after invasive VNS in humans (El Tahry et al. 2010; Evans et al. 2004; Koo et al. 2001) or electrophysiological recordings from the vagus nerve in anesthetized rats (Kuo et al. 2005). Notably, another study using invasive vagus nerve recordings challenged this assumption, demonstrating that direct measures of vagal activity are not associated with HRV (measured SDRR, RMSSD, HF‐HRV, and LF/HF ratio) in anesthetized and behaving rats (Marmerstein et al. 2021). Given the end‐organ specificity of vagal fibers, it is conceivable that the effects of VNS vary depending on which specific fibers are stimulated (Evans et al. 2004; Jayaprakash et al. 2023; Patros, Farmer, Moneghetti, et al. 2024; Qing et al. 2018). Hence, the idea of a unitary vagal activity is likely to be an oversimplification (Grossman 2023; Teckentrup and Kroemer 2024). Relatedly, it has been proposed that HRV is associated with signals from cardiac vagal efferents and that, specifically, HF‐HRV reflects the cardiac vagal modulations and baroreceptor afferent signaling (Eckberg 1983; Grossman and Taylor 2007; Hedman et al. 1995). Whereas tonic decreases in HRV may reflect impairments in the body's autonomic function (Coopmans et al. 2020; Laborde et al. 2017; Thayer and Lane 2007), an acute (phasic) decrease in HRV can be adaptive, reflecting an individual's capacity to allocate energy to cope with demand (Dickerson and Kemeny 2004; Thayer et al. 2009). Intriguingly, such demand may correspond with vagal afferent signals affecting arousal and motivational drive (Chen et al. 2023; D'Agostini et al. 2023; Neuser et al. 2020; Rong et al. 2014). As HF‐HRV and respiration are closely linked (Grossman and Taylor 2007; Jensen et al. 2022; Soer et al. 2021), we examined changes in respiratory rate following stimulation and found no significant difference between taVNS and sham. Our findings indicate that variations in respiratory rate did not confound our main outcome related to taVNS effects.

Notably, taVNS studies on any HRV indices yielded mixed results. In contrast to our findings, several studies reported increases (SDNN: De Couck et al. 2017; RMSSD, HF‐HRV & SDRR: Forte et al. 2022; LF/HF ratio: Gauthey et al. 2020; RMSSD, HF‐HRV & SDRR: Geng, Liu, et al. 2022; RMSSD: Geng, Yang, et al. 2022; SDNN, RMSSD & pNN50: Machetanz et al. 2021), or no significant change in HRV (Borges et al. 2019; Burger et al. 2019; Šinkovec et al. 2023; Villani et al. 2019), leading to non‐significant taVNS‐induced changes across published studies so far (Wolf et al. 2021). It is conceivable that differences in study design, sample characteristics, taVNS protocols, and time of day (i.e., circadian effects) contribute to heterogeneity across studies. For instance, out of the studies showing a decrease in HRV during taVNS, Altınkaya et al. (2023) was the only study with a comparable design, albeit with a smaller sample size (*n* = 14). As the optimal stimulation protocol is unknown, it is essential to examine taVNS‐induced changes across well‐designed, randomized, sham‐controlled, and within‐subject designs to guide future research. Consequently, we find consistent effects across complementary indices of HRV (RMSSD, HF‐HRV, and SDRR), physiological states, and sides of the stimulation, effectively replicating taVNS‐induced changes from independent sessions within the study.

An important factor that might modulate the effectiveness of taVNS in regulating cardiovascular function is the body's metabolic state (Kozorosky et al. 2022; Sclocco et al. 2019; Szulczewski 2022). After the caloric load, we found that taVNS decreased HF‐HRV and RMSSD, further amplifying the reduction in RMSSD and HF‐HRV induced by the milkshake. Notably, while SDRR and LF/HF ratio increased after the caloric load, taVNS still reduced SDRR, with no effect on the LF/HF ratio. Likewise, other studies have reported either a decrease in HF‐HRV after a caloric load (Lu et al. 1999; Ohara et al. 2015) or an increase in LF/HF ratio after an oral glucose tolerance test (Vosseler et al. 2020). Early stages of digestion may be linked to withdrawal of cardiac vagal activity, inhibiting the heart's pacemaker (Ng et al. 2001) and resulting in an increased HR. Vagally mediated processes are crucial to calibrate energy metabolism after increases in glucose levels to regulate eating behavior according to metabolic demands (Berthoud 2008; Berthoud et al. 2021; Prescott and Liberles 2022). Of note, two previous studies reported non‐significant or inconclusive taVNS‐induced changes in RMSSD after administering a caloric load of ~100 kcal less than in our study to fasted participants (Altınkaya et al. 2023; Vosseler et al. 2020) using a comparable stimulation protocol. Since these studies were considerably smaller (*N*s = 14 and 15), these differences could be mostly due to power (i.e., increasing *N* to 36 improves power for moderate within‐subject effects *dz* ~0.6 from 58% to 94%). We conclude that taVNS decreased SDRR, RMSSD, and HF‐HRV before and after the caloric load across stimulation sides, even though the caloric load had opposing effects on HRV indices, indicating that taVNS‐induced changes are largely independent of metabolic state.

Crucially, our study also provides much‐needed information regarding the debate about the side‐specific effects of taVNS (Kim et al. 2022) on cardiovascular function, which have important implications for its safety. In contrast to concerns about potential increases in the risk for bradycardia due to cardiac vagal hyperstimulation (e.g., due to a case report for long‐term VNS stimulation: Pascual 2015; Shankar et al. 2013), we only found support for acutely induced reductions in HRV (RMSSD, SDRR, and HF‐HRV), not HR. In general, reports of adverse cardiac effects in healthy young individuals after right taVNS and even VNS (Patros, Farmer, Ottaviani, et al. 2024) are scarce and do not support a clinically relevant side‐specific modulation (Redgrave et al. 2018). Capitalizing on our within‐subject design, we quantitatively examined the hypothesized lateralization of taVNS‐induced changes in HR and HRV (RMSSD, HF‐HRV, SDRR, and LF/HF ratio). We showed that taVNS‐induced changes in HR and HRV are comparable across sides, providing conclusive evidence against large side‐specific effects. While previous studies have reported an increase in SDNN with right cymba taVNS compared to the left side (De Couck et al. 2017; Machetanz et al. 2021), the former used no stimulation as a reference (De Couck et al. 2017), and the latter found the same direction of the effect on RMSSD, pNN50, SD1, and SD2, but used a cavum conchae sham in a between‐subject design (Machetanz et al. 2021). Moreover, we found that taVNS‐induced changes in HR and HRV (RMSSD, HF‐HRV, SDRR, and LF/HF ratio) between sides were more similar compared to sham‐induced changes, indicating that they likely recruited a comparable efferent pathway. While participants reported no adverse events after taVNS in our study, additional studies monitoring safety during taVNS at the right ear are required to validate our findings. Taken together, our findings demonstrate that taVNS elicits modulatory effects on the cardiovascular system that are comparable on both sides, suggesting that right taVNS can be used as safely as left taVNS.

Our study found that stimulation per se decreased HR, regardless of condition. Other studies reported reduced HR during taVNS in healthy individuals (Antonino et al. 2017; Badran et al. 2018; Clancy et al. 2014; Paleczny et al. 2021), whereas no changes were observed during invasive VNS (Barone et al. 2007; Kamath et al. 1992; Ronkainen et al. 2006) or taVNS compared to sham at the ear lobe (e.g., Gauthey et al. 2020; Vosseler et al. 2020). It is conceivable that the great auricular nerve stimulation may lead to comparable effects on HR (Cakmak et al. 2018), suggesting that the induced changes are not specific to taVNS. Alternatively, it may reflect gradual changes in the state of the participants relative to the baseline.

Despite producing converging results across physiological states and stimulation sides, our taVNS study has limitations. First, we used the conventional continuous stimulation protocol and pulsed taVNS (Keute et al. 2021), or low‐intensity settings (Šinkovec et al. 2023), which may exert different effects on cardiovascular function. Second, future studies could benefit from incorporating additional biomarkers, such as salivary alpha‐amylase (Bach 2014; Giraudier et al. 2022), pupil diameter (D'Agostini et al. 2023; Ludwig et al. 2024; Pervaz et al. 2024), or muscle sympathetic nerve activity (Clancy et al. 2014), that provide complementary information on the activity of the sympathetic or parasympathetic system. Such biomarkers may support the interpretation of taVNS‐induced effects on HRV (see Clancy et al. 2014; Koenig et al. 2021), thereby advancing our understanding of body–brain regulation (Bates et al. 2023).

To conclude, the vagus nerve plays a crucial role in regulating the cardiovascular system according to demand. Here, we investigated the effects of taVNS on HR, HRV (SDRR, RMSSD, HF‐HRV, and HF/LF ratio), and respiration rate in a randomized crossover design, considering potential interactions with the side of the stimulation and metabolic states (i.e., before and after consuming a milkshake). Our results revealed no taVNS‐induced changes in HR, while HRV, as indexed by HF‐HRV, RMSSD, and SDRR, decreased during stimulation and after the caloric load. The decrease in HRV as indexed by HF‐HRV, RMSSD, and SDRR due to stimulating vagal afferents contradicts the assumption that stimulation of the vagus nerve per se increases cardiovagal activity (El Tahry et al. 2010; Evans et al. 2004; Koo et al. 2001; Patros et al. 2022) and would lead to an increase in HF‐HRV or RMSSD by its indirect effect on the autonomic nervous system. Our results add to the mixed meta‐analytic findings (Wolf et al. 2021) and suggest that cardiovascular effects might be best contextualized with VNS‐induced increases in arousal and motivational drive instead of relaxation (Teckentrup and Kroemer 2024). Moreover, we conclude that taVNS‐induced effects on the cardiovascular system can be elicited on both sides and in different metabolic states with similar effectiveness without reducing HR or increasing the risk of bradycardia and adverse events in healthy participants. Consequently, future applications targeting potentially lateralized modulatory effects of taVNS can likely stimulate the right auricular branch of the vagus nerve as safely as the left branch.

## Author Contributions

**Kristin Kaduk:** formal analysis, visualization, writing – original draft, writing – review and editing. **Alessandro Petrella:** investigation, methodology, project administration, writing – review and editing. **Sophie J. Müller:** investigation, methodology, project administration, writing – review and editing. **Julian Koenig:** writing – review and editing. **Nils B. Kroemer:** conceptualization, funding acquisition, resources, supervision, validation, writing – original draft, writing – review and editing.

## Conflicts of Interest

J.K. has collaborative third‐party funding and received honoraria by tVNS Technologies, Erlangen Germany. All other authors declare no conflicts of interest.

## Supporting information


**Data S1.**.

## Data Availability

Raw data from the current study are available on Open Science Framework (https://osf.io/26v5n).

## References

[psyp70017-bib-0001] Akselrod, S. , D. Gordon , F. A. Ubel , D. C. Shannon , A. C. Berger , and R. J. Cohen . 1981. “Power Spectrum Analysis of Heart Rate Fluctuation: A Quantitative Probe of Beat‐To‐Beat Cardiovascular Control.” Science 213, no. 4504: 220–222. 10.1126/science.6166045. 6166045

[psyp70017-bib-0002] Allen, M. , A. Levy , T. Parr , and K. J. Friston . 2022. “In the Body's Eye: The Computational Anatomy of Interoceptive Inference.” PLoS Computational Biology 18, no. 9: e1010490. 10.1371/journal.pcbi.1010490. 36099315 PMC9506608

[psyp70017-bib-0003] Altınkaya, Z. , L. Öztürk , İ. Büyükgüdük , et al. 2023. “Non‐invasive Vagus Nerve Stimulation in a Hungry State Decreases Heart Rate Variability.” Physiology & Behavior 258: 114016. 10.1016/j.physbeh.2022.114016. 36334796

[psyp70017-bib-0004] Ambarish, V. , P. Barde , A. Vyas , and K. K. Deepak . 2005. “Comparison Between Pre‐Prandial and Post‐Prandial Heart Rate Variability (HRV).” Indian Journal of Physiology and Pharmacology 49, no. 4: 436–442. 16579397

[psyp70017-bib-0005] Anselmi, L. , L. Toti , C. Bove , and R. A. Travagli . 2017. “Vagally Mediated Effects of Brain Stem Dopamine on Gastric Tone and Phasic Contractions of the Rat.” American Journal of Physiology: Gastrointestinal and Liver Physiology 313, no. 5: G434–G441. 10.1152/ajpgi.00180.2017. 28729246 PMC5792220

[psyp70017-bib-0006] Antonino, D. , A. L. Teixeira , P. M. Maia‐Lopes , et al. 2017. “Non‐invasive Vagus Nerve Stimulation Acutely Improves Spontaneous Cardiac Baroreflex Sensitivity in Healthy Young Men: A Randomized Placebo‐Controlled Trial.” Brain Stimulation 10, no. 5: 875–881. 10.1016/j.brs.2017.05.006. 28566194

[psyp70017-bib-0007] Ardell, J. L. , and W. C. Randall . 1986. “Selective Vagal Innervation of Sinoatrial and Atrioventricular Nodes in Canine Heart.” American Journal of Physiology—Heart and Circulatory Physiology 251, no. 4: H764–H773. 10.1152/ajpheart.1986.251.4.H764. 3021001

[psyp70017-bib-0008] Armstrong, R. , P. Wheen , L. Brandon , A. Maree , and R.‐A. Kenny . 2022. “Heart Rate: Control Mechanisms, Pathophysiology and Assessment of the Neurocardiac System in Health and Disease.” Quarterly Journal of Medicine 115, no. 12: 806–812. 10.1093/qjmed/hcab016. 33486521

[psyp70017-bib-0009] Bach, D. R. 2014. “Sympathetic Nerve Activity Can Be Estimated From Skin Conductance Responses—A Comment on Henderson Et al. (2012).” NeuroImage 84: 122–123. 10.1016/j.neuroimage.2013.08.030. 23994456 PMC3898862

[psyp70017-bib-0010] Badran, B. W. , O. J. Mithoefer , C. E. Summer , et al. 2018. “Short Trains of Transcutaneous Auricular Vagus Nerve Stimulation (taVNS) Have Parameter‐Specific Effects on Heart Rate.” Brain Stimulation 11, no. 4: 699–708. 10.1016/j.brs.2018.04.004. 29716843 PMC6536129

[psyp70017-bib-0011] Barone, L. , G. Colicchio , D. Policicchio , et al. 2007. “Effect of Vagal Nerve Stimulation on Systemic Inflammation and Cardiac Autonomic Function in Patients With Refractory Epilepsy.” Neuroimmunomodulation 14, no. 6: 331–336. 10.1159/000127360. 18418007

[psyp70017-bib-0012] Bates, M. E. , D. Eddie , P. M. Lehrer , R. P. Nolan , and M. Siepmann . 2023. “Editorial: Integrated Cardiovascular and Neural System Processes as Potential Mechanisms of Behavior Change.” Frontiers in Psychiatry 14: 1175691. 10.3389/fpsyt.2023.1175691. 37032946 PMC10074486

[psyp70017-bib-0013] Berthoud, H.‐R. 2008. “The Vagus Nerve, Food Intake and Obesity.” Regulatory Peptides 149, no. 1–3: 15–25. 10.1016/j.regpep.2007.08.024. 18482776 PMC2597723

[psyp70017-bib-0014] Berthoud, H.‐R. , V. L. Albaugh , and W. L. Neuhuber . 2021. “Gut‐Brain Communication and Obesity: Understanding Functions of the Vagus Nerve.” Journal of Clinical Investigation 131, no. 10: e143770. 10.1172/JCI143770. 33998597 PMC8121510

[psyp70017-bib-0015] Billman, G. E. 2013. “The LF/HF Ratio Does Not Accurately Measure Cardiac Sympatho‐Vagal Balance.” Frontiers in Physiology 4: 26. 10.3389/fphys.2013.00026. 23431279 PMC3576706

[psyp70017-bib-0016] Borges, U. , S. Laborde , and M. Raab . 2019. “Influence of Transcutaneous Vagus Nerve Stimulation on Cardiac Vagal Activity: Not Different From Sham Stimulation and no Effect of Stimulation Intensity.” PLoS One 14, no. 10: e0223848. 10.1371/journal.pone.0223848. 31603939 PMC6788680

[psyp70017-bib-0017] Borgmann, D. , L. Rigoux , B. Kuzmanovic , et al. 2021. “Technical Note: Modulation of fMRI Brainstem Responses by Transcutaneous Vagus Nerve Stimulation.” NeuroImage 244: 118566. 10.1016/j.neuroimage.2021.118566. 34509623

[psyp70017-bib-0018] Boyett, M. 2000. “The Sinoatrial Node, a Heterogeneous Pacemaker Structure.” Cardiovascular Research 47, no. 4: 658–687. 10.1016/S0008-6363(00)00135-8. 10974216

[psyp70017-bib-0019] Bretherton, B. , L. Atkinson , A. Murray , J. Clancy , S. Deuchars , and J. Deuchars . 2019. “Effects of Transcutaneous Vagus Nerve Stimulation in Individuals Aged 55 Years or Above: Potential Benefits of Daily Stimulation.” Aging 11, no. 14: 4836–4857. 10.18632/aging.102074. 31358702 PMC6682519

[psyp70017-bib-0020] Brougher, J. , U. Aziz , N. Adari , et al. 2021. “Self‐Administration of Right Vagus Nerve Stimulation Activates Midbrain Dopaminergic Nuclei.” Frontiers in Neuroscience 15: 782786. 10.3389/fnins.2021.782786. 34975384 PMC8716493

[psyp70017-bib-0021] Burger, A. M. , M. D'Agostini , B. Verkuil , and I. Van Diest . 2020. “Moving Beyond Belief: A Narrative Review of Potential Biomarkers for Transcutaneous Vagus Nerve Stimulation.” Psychophysiology 57, no. 6: e13571. 10.1111/psyp.13571. 32202671

[psyp70017-bib-0022] Burger, A. M. , W. Van der Does , J. F. Thayer , J. F. Brosschot , and B. Verkuil . 2019. “Transcutaneous Vagus Nerve Stimulation Reduces Spontaneous but Not Induced Negative Thought Intrusions in High Worriers.” Biological Psychology 142: 80–89. 10.1016/j.biopsycho.2019.01.014. 30710565

[psyp70017-bib-0023] Butt, M. F. , A. Albusoda , A. D. Farmer , and Q. Aziz . 2020. “The Anatomical Basis for Transcutaneous Auricular Vagus Nerve Stimulation.” Journal of Anatomy 236, no. 4: 588–611. 10.1111/joa.13122. 31742681 PMC7083568

[psyp70017-bib-0024] Cakmak, Y. O. , S. Cotofana , C. Jäger , et al. 2018. “Peri‐Arterial Autonomic Innervation of the Human Ear.” Scientific Reports 8, no. 1: 11469. 10.1038/s41598-018-29839-z. 30065349 PMC6068185

[psyp70017-bib-0025] Capilupi, M. J. , S. M. Kerath , and L. B. Becker . 2020. “Vagus Nerve Stimulation and the Cardiovascular System.” Cold Spring Harbor Perspectives in Medicine 10, no. 2: a034173. 10.1101/cshperspect.a034173. 31109966 PMC6996447

[psyp70017-bib-0026] Chapman, C. L. , E. L. Reed , M. L. Worley , et al. 2021. “Sugar‐Sweetened Soft Drink Consumption Acutely Decreases Spontaneous Baroreflex Sensitivity and Heart Rate Variability.” American Journal of Physiology—Regulatory, Integrative and Comparative Physiology 320, no. 5: R641–R652. 10.1152/ajpregu.00310.2020. 33533320 PMC8163604

[psyp70017-bib-0027] Chen, M. , L. Yu , F. Ouyang , et al. 2015. “The Right Side or Left Side of Noninvasive Transcutaneous Vagus Nerve Stimulation: Based on Conventional Wisdom or Scientific Evidence?” International Journal of Cardiology 187: 44–45. 10.1016/j.ijcard.2015.03.351. 25828310

[psyp70017-bib-0028] Chen, Y. , X. Lu , and L. Hu . 2023. “Transcutaneous Auricular Vagus Nerve Stimulation Facilitates Cortical Arousal and Alertness.” International Journal of Environmental Research and Public Health 20, no. 2: 1402. 10.3390/ijerph20021402. 36674156 PMC9859411

[psyp70017-bib-0029] Clancy, J. A. , D. A. Mary , K. K. Witte , J. P. Greenwood , S. A. Deuchars , and J. Deuchars . 2014. “Non‐invasive Vagus Nerve Stimulation in Healthy Humans Reduces Sympathetic Nerve Activity.” Brain Stimulation 7, no. 6: 871–877. 10.1016/j.brs.2014.07.031. 25164906

[psyp70017-bib-0030] Coopmans, C. , T. L. Zhou , R. M. A. Henry , et al. 2020. “Both Prediabetes and Type 2 Diabetes Are Associated With Lower Heart Rate Variability: The Maastricht Study.” Diabetes Care 43, no. 5: 1126–1133. 10.2337/dc19-2367. 32161051

[psyp70017-bib-0031] D'Agostini, M. , A. M. Burger , M. Franssen , et al. 2023. “Short Bursts of Transcutaneous Auricular Vagus Nerve Stimulation Enhance Evoked Pupil Dilation as a Function of Stimulation Parameters.” Cortex 159: 233–253. 10.1016/j.cortex.2022.11.012. 36640622

[psyp70017-bib-0032] De Couck, M. , R. Cserjesi , R. Caers , et al. 2017. “Effects of Short and Prolonged Transcutaneous Vagus Nerve Stimulation on Heart Rate Variability in Healthy Subjects.” Autonomic Neuroscience 203: 88–96. 10.1016/j.autneu.2016.11.003. 28017263

[psyp70017-bib-0033] Dickerson, S. S. , and M. E. Kemeny . 2004. “Acute Stressors and Cortisol Responses: A Theoretical Integration and Synthesis of Laboratory Research.” Psychological Bulletin 130, no. 3: 355–391. 10.1037/0033-2909.130.3.355. 15122924

[psyp70017-bib-0034] Eckberg, D. L. 1983. “Human Sinus Arrhythmia as an Index of Vagal Cardiac Outflow.” Journal of Applied Physiology 54, no. 4: 961–966. 10.1152/jappl.1983.54.4.961. 6853303

[psyp70017-bib-0035] El Tahry, R. , R. Raedt , L. Mollet , et al. 2010. “A Novel Implantable Vagus Nerve Stimulation System (ADNS‐300) for Combined Stimulation and Recording of the Vagus Nerve: Pilot Trial at Ghent University Hospital.” Epilepsy Research 92, no. 2–3: 231–239. 10.1016/j.eplepsyres.2010.10.007. 21071177

[psyp70017-bib-0036] Evans, M. S. , S. Verma‐Ahuja , D. K. Naritoku , and J. A. Espinosa . 2004. “Intraoperative Human Vagus Nerve Compound Action Potentials.” Acta Neurologica Scandinavica 110, no. 4: 232–238. 10.1111/j.1600-0404.2004.00309.x. 15355486

[psyp70017-bib-0037] Farmer, A. D. , A. Strzelczyk , A. Finisguerra , et al. 2021. “International Consensus Based Review and Recommendations for Minimum Reporting Standards in Research on Transcutaneous Vagus Nerve Stimulation (Version 2020).” Frontiers in Human Neuroscience 14: 568051. 10.3389/fnhum.2020.568051. 33854421 PMC8040977

[psyp70017-bib-0038] Faul, F. , E. Erdfelder , A.‐G. Lang , and A. Buchner . 2007. “G*Power 3: A Flexible Statistical Power Analysis Program for the Social, Behavioral, and Biomedical Sciences.” Behavior Research Methods 39, no. 2: 175–191. 10.3758/BF03193146. 17695343

[psyp70017-bib-0039] Ferstl, M. , V. Teckentrup , W. M. Lin , et al. 2021. “Non‐invasive Vagus Nerve Stimulation Boosts Mood Recovery After Effort Exertion.” Psychological Medicine 52, no. 14: 1–11. 10.1017/S0033291720005073. PMC9693679 33586647

[psyp70017-bib-0040] Forte, G. , F. Favieri , E. Leemhuis , et al. 2022. “Ear Your Heart: Transcutaneous Auricular Vagus Nerve Stimulation on Heart Rate Variability in Healthy Young Participants.” PeerJ 10: e14447. 10.7717/peerj.14447. 36438582 PMC9686410

[psyp70017-bib-0041] Frangos, E. , J. Ellrich , and B. R. Komisaruk . 2015. “Non‐invasive Access to the Vagus Nerve Central Projections via Electrical Stimulation of the External Ear: fMRI Evidence in Humans.” Brain Stimulation 8, no. 3: 624–636. 10.1016/j.brs.2014.11.018. 25573069 PMC4458242

[psyp70017-bib-0042] Gauthey, A. , S. Morra , P. van de Borne , D. Deriaz , N. Maes , and J.‐B. le Polain de Waroux . 2020. “Sympathetic Effect of Auricular Transcutaneous Vagus Nerve Stimulation on Healthy Subjects: A Crossover Controlled Clinical Trial Comparing Vagally Mediated and Active Control Stimulation Using Microneurography.” Frontiers in Physiology 11: 599896. 10.3389/fphys.2020.599896. 33343394 PMC7744823

[psyp70017-bib-0043] Geng, D. , X. Liu , Y. Wang , and J. Wang . 2022. “The Effect of Transcutaneous Auricular Vagus Nerve Stimulation on HRV in Healthy Young People.” PLoS One 17, no. 2: e0263833. 10.1371/journal.pone.0263833. 35143576 PMC8830655

[psyp70017-bib-0044] Geng, D. , K. Yang , Z. Fu , Y. Zhang , C. Wang , and H. An . 2022. “Circadian Stage‐Dependent and Stimulation Duration Effects of Transcutaneous Auricular Vagus Nerve Stimulation on Heart Rate Variability.” PLoS One 17, no. 11: e0277090. 10.1371/journal.pone.0277090. 36327249 PMC9632923

[psyp70017-bib-0045] Giraudier, M. , C. Ventura‐Bort , A. M. Burger , et al. 2022. “Evidence for a Modulating Effect of Transcutaneous Auricular Vagus Nerve Stimulation (taVNS) on Salivary Alpha‐Amylase as Indirect Noradrenergic Marker: A Pooled Mega‐Analysis.” Brain Stimulation 15, no. 6: 1378–1388. 10.1016/j.brs.2022.09.009. 36183953

[psyp70017-bib-0046] Gourine, A. V. , A. Machhada , S. Trapp , and K. M. Spyer . 2016. “Cardiac Vagal Preganglionic Neurones: An Update.” Autonomic Neuroscience 199: 24–28. 10.1016/j.autneu.2016.06.003. 27396874

[psyp70017-bib-0047] Green, J. A. 2011. “The Heart Rate Method for Estimating Metabolic Rate: Review and Recommendations.” Comparative Biochemistry and Physiology Part A: Molecular & Integrative Physiology 158, no. 3: 287–304. 10.1016/j.cbpa.2010.09.011. 20869457

[psyp70017-bib-0048] Grossman, P. 2023. “Fundamental Challenges and Likely Refutations of the Five Basic Premises of the Polyvagal Theory.” Biological Psychology 180: 108589. 10.1016/j.biopsycho.2023.108589. 37230290

[psyp70017-bib-0049] Grossman, P. , and E. W. Taylor . 2007. “Toward Understanding Respiratory Sinus Arrhythmia: Relations to Cardiac Vagal Tone, Evolution and Biobehavioral Functions.” Biological Psychology 74, no. 2: 263–285. 10.1016/j.biopsycho.2005.11.014. 17081672

[psyp70017-bib-0050] Han, W. , L. A. Tellez , M. H. Perkins , et al. 2018. “A Neural Circuit for Gut‐Induced Reward.” Cell 175, no. 3: 665–678. 10.1016/j.cell.2018.08.049. 30245012 PMC6195474

[psyp70017-bib-0051] Havel, P. J. 2001. “Peripheral Signals Conveying Metabolic Information to the Brain: Short‐Term and Long‐Term Regulation of Food Intake and Energy Homeostasis.” Experimental Biology and Medicine 226, no. 11: 963–977. 10.1177/153537020122601102. 11743131

[psyp70017-bib-0052] Hedman, A. E. , J. E. K. Hartikainen , K. U. O. Tahvanainen , and M. O. K. Hakumäki . 1995. “The High Frequency Component of Heart Rate Variability Reflects Cardiac Parasympathetic Modulation Rather Than Parasympathetic ‘Tone’.” Acta Physiologica Scandinavica 155, no. 3: 267–273. 10.1111/j.1748-1716.1995.tb09973.x. 8619324

[psyp70017-bib-0053] Heni, M. , R. Wagner , S. Kullmann , et al. 2014. “Central Insulin Administration Improves Whole‐Body Insulin Sensitivity via Hypothalamus and Parasympathetic Outputs in Men.” Diabetes Care 63, no. 12: 4083–4088. 10.2337/db14-0477. 25028522

[psyp70017-bib-0054] Hong, G.‐S. , B. Pintea , P. Lingohr , et al. 2019. “Effect of Transcutaneous Vagus Nerve Stimulation on Muscle Activity in the Gastrointestinal Tract (transVaGa): A Prospective Clinical Trial.” International Journal of Colorectal Disease 34, no. 3: 417–422. 10.1007/s00384-018-3204-6. 30519842

[psyp70017-bib-0055] Huang, J. , Y. Wang , D. Jiang , J. Zhou , and X. Huang . 2010. “The Sympathetic‐Vagal Balance Against Endotoxemia.” Journal of Neural Transmission 117, no. 6: 729–735. 10.1007/s00702-010-0407-6. 20458507

[psyp70017-bib-0056] Jayaprakash, N. , W. Song , V. Toth , et al. 2023. “Organ‐ and Function‐Specific Anatomical Organization of Vagal Fibers Supports Fascicular Vagus Nerve Stimulation.” Brain Stimulation 16, no. 2: 484–506. 10.1016/j.brs.2023.02.003. 36773779 PMC10228508

[psyp70017-bib-0057] Jeffreys, H. 1939. Theory of Probability. Oxford University Press. 10.1093/oso/9780198503682.001.0001.

[psyp70017-bib-0058] Jensen, M. K. , S. S. Andersen , S. S. Andersen , C. H. Liboriussen , S. Kristensen , and M. Jochumsen . 2022. “Modulating Heart Rate Variability Through Deep Breathing Exercises and Transcutaneous Auricular Vagus Nerve Stimulation: A Study in Healthy Participants and in Patients With Rheumatoid Arthritis or Systemic Lupus Erythematosus.” Sensors 22, no. 20: 7884. 10.3390/s22207884. 36298234 PMC9607552

[psyp70017-bib-0059] Kamath, M. V. , A. R. M. Upton , A. Talalla , and E. L. Fallen . 1992. “Neurocardiac Responses to Vagoafferent Electrostimulation in Humans.” Pacing and Clinical Electrophysiology 15, no. 10: 1581–1587. 10.1111/j.1540-8159.1992.tb02937.x. 1383973

[psyp70017-bib-0060] Kay, G. N. , M. S. Ashar , R. S. Bubien , and S. M. Dailey . 1995. “Relationship Between Heart Rate and Oxygen Kinetics During Constant Workload Exercise.” Pacing and Clinical Electrophysiology 18, no. 10: 1853–1860. 10.1111/j.1540-8159.1995.tb03832.x. 8539152

[psyp70017-bib-0061] Keute, M. , K. Machetanz , L. Berelidze , R. Guggenberger , and A. Gharabaghi . 2021. “Neuro‐Cardiac Coupling Predicts Transcutaneous Auricular Vagus Nerve Stimulation Effects.” Brain Stimulation 14, no. 2: 209–216. 10.1016/j.brs.2021.01.001. 33422683

[psyp70017-bib-0062] Kim, A. Y. , A. Marduy , P. S. de Melo , et al. 2022. “Safety of Transcutaneous Auricular Vagus Nerve Stimulation (taVNS): A Systematic Review and Meta‐Analysis.” Scientific Reports 12, no. 1: 22055. 10.1038/s41598-022-25864-1. 36543841 PMC9772204

[psyp70017-bib-0063] Koenig, J. , P. Parzer , N. Haigis , et al. 2021. “Effects of Acute Transcutaneous Vagus Nerve Stimulation on Emotion Recognition in Adolescent Depression.” Psychological Medicine 51, no. 3: 511–520. 10.1017/S0033291719003490. 31818339 PMC7958483

[psyp70017-bib-0064] Koo, B. , S. D. Ham , S. Sood , and B. Tarver . 2001. “Human Vagus Nerve Electrophysiology: A Guide to Vagus Nerve Stimulation Parameters.” Journal of Clinical Neurophysiology 18, no. 5: 429–433. 10.1097/00004691-200109000-00007. 11709648

[psyp70017-bib-0065] Kozorosky, E. M. , C. H. Lee , J. G. Lee , V. Nunez Martinez , L. E. Padayachee , and H. M. Stauss . 2022. “Transcutaneous Auricular Vagus Nerve Stimulation Augments Postprandial Inhibition of Ghrelin.” Physiological Reports 10, no. 8: e15253. 10.14814/phy2.15253. 35441808 PMC9020171

[psyp70017-bib-0066] Kuo, T. B. J. , C. J. Lai , Y. Huang , and C. C. H. Yang . 2005. “Regression Analysis Between Heart Rate Variability and Baroreflex‐Related Vagus Nerve Activity in Rats.” Journal of Cardiovascular Electrophysiology 16, no. 8: 864–869. 10.1111/j.1540-8167.2005.40656.x. 16101628

[psyp70017-bib-0067] Laborde, S. , E. Mosley , and J. F. Thayer . 2017. “Heart Rate Variability and Cardiac Vagal Tone in Psychophysiological Research—Recommendations for Experiment Planning, Data Analysis, and Data Reporting.” Frontiers in Psychology 8: 213. 10.3389/fpsyg.2017.00213. 28265249 PMC5316555

[psyp70017-bib-0068] Lee, S. W. , A. Anderson , P. A. Guzman , A. Nakano , E. G. Tolkacheva , and K. Wickman . 2018. “Atrial GIRK Channels Mediate the Effects of Vagus Nerve Stimulation on Heart Rate Dynamics and Arrhythmogenesis.” Frontiers in Physiology 9: 943. 10.3389/fphys.2018.00943. 30072916 PMC6060443

[psyp70017-bib-0069] Lewine, J. D. , K. Paulson , N. Bangera , and B. J. Simon . 2019. “Exploration of the Impact of Brief Noninvasive Vagal Nerve Stimulation on EEG and Event‐Related Potentials.” Neuromodulation: Technology at the Neural Interface 22, no. 5: 564–572. 10.1111/ner.12864. 30288866

[psyp70017-bib-0070] Lindmark, S. , U. Wiklund , P. Bjerle , and J. W. Eriksson . 2003. “Does the Autonomic Nervous System Play a Role in the Development of Insulin Resistance? A Study on Heart Rate Variability in First‐Degree Relatives of Type 2 Diabetes Patients and Control Subjects: Original Article.” Diabetic Medicine 20, no. 5: 399–405. 10.1046/j.1464-5491.2003.00920.x. 12752490

[psyp70017-bib-0071] Lloyd, B. , F. Wurm , R. De Kleijn , and S. Nieuwenhuis . 2023. “Short‐Term Transcutaneous Vagus Nerve Stimulation Increases Pupil Size but Does Not Affect EEG Alpha Power: A Replication.” Neuroscience. 10.1101/2023.03.08.531479. 37348704

[psyp70017-bib-0072] Lu, C. L. , X. Zou , W. C. Orr , and J. D. Chen . 1999. “Postprandial Changes of Sympathovagal Balance Measured by Heart Rate Variability.” Digestive Diseases and Sciences 44, no. 4: 857–861. 10.1023/a:1026698800742. 10219849

[psyp70017-bib-0073] Ludwig, M. , C. Pereira , M. Keute , E. Düzel , M. J. Betts , and D. Hämmerer . 2024. “Evaluating Phasic Transcutaneous Vagus Nerve Stimulation (taVNS) With Pupil Dilation: The Importance of Stimulation Intensity and Sensory Perception.” Scientific Reports 14, no. 1: 24391. 10.1038/s41598-024-72179-4. 39420188 PMC11487125

[psyp70017-bib-0074] Machetanz, K. , L. Berelidze , R. Guggenberger , and A. Gharabaghi . 2021. “Transcutaneous Auricular Vagus Nerve Stimulation and Heart Rate Variability: Analysis of Parameters and Targets.” Autonomic Neuroscience 236: 102894. 10.1016/j.autneu.2021.102894. 34662844

[psyp70017-bib-0075] Makowski, D. , T. Pham , Z. J. Lau , et al. 2021. “NeuroKit2: A Python Toolbox for Neurophysiological Signal Processing.” Behavior Research Methods 53, no. 4: 1689–1696. 10.3758/s13428-020-01516-y. 33528817

[psyp70017-bib-0076] Malik, M. , and A. J. Camm . 1993. “Components of Heart Rate Variability—What They Really Mean and What We Really Measure.” American Journal of Cardiology 72, no. 11: 821–822. 10.1016/0002-9149(93)91070-X. 8093124

[psyp70017-bib-0077] Maniscalco, J. W. , and L. Rinaman . 2018. “Vagal Interoceptive Modulation of Motivated Behavior.” Physiology 33, no. 2: 151–167. 10.1152/physiol.00036.2017. 29412062 PMC5899236

[psyp70017-bib-0078] Marmerstein, J. T. , G. A. McCallum , and D. M. Durand . 2021. “Direct Measurement of Vagal Tone in Rats Does Not Show Correlation to HRV.” Scientific Reports 11, no. 1: 1210. 10.1038/s41598-020-79808-8. 33441733 PMC7807082

[psyp70017-bib-0079] Meule, A. , T. Hermann , and A. Kübler . 2014. “A Short Version of the Food Cravings Questionnaire Trait: The FCQ‐T‐Reduced.” Frontiers in Psychology 5: 190. 10.3389/fpsyg.2014.00190. 24624116 PMC3940888

[psyp70017-bib-0080] Nagai, N. , N. Sakane , and T. Moritani . 2005. “Metabolic Responses to High‐Fat or Low‐Fat Meals and Association With Sympathetic Nervous System Activity in Healthy Young Men.” Journal of Nutritional Science and Vitaminology 51, no. 5: 355–360. 10.3177/jnsv.51.355. 16392707

[psyp70017-bib-0081] Neuser, M. P. , V. Teckentrup , A. Kühnel , M. Hallschmid , M. Walter , and N. B. Kroemer . 2020. “Vagus Nerve Stimulation Boosts the Drive to Work for Rewards.” Nature Communications 11, no. 1: 3555. 10.1038/s41467-020-17344-9. PMC7366927 32678082

[psyp70017-bib-0082] Ng, G. A. , K. E. Brack , and J. H. Coote . 2001. “Effects of Direct Sympathetic and Vagus Nerve Stimulation on the Physiology of the Whole Heart—A Novel Model of Isolated Langendorff Perfused Rabbit Heart With Intact Dual Autonomic Innervation.” Experimental Physiology 86, no. 3: 319–329. 10.1113/eph8602146. 11471534

[psyp70017-bib-0083] Nummenmaa, L. , E. Glerean , R. Hari , and J. K. Hietanen . 2014. “Bodily Maps of Emotions.” Proceedings of the National Academy of Sciences of the United States of America 111, no. 2: 646–651. 10.1073/pnas.1321664111. 24379370 PMC3896150

[psyp70017-bib-0084] Ohara, K. , Y. Okita , K. Kouda , T. Mase , C. Miyawaki , and H. Nakamura . 2015. “Cardiovascular Response to Short‐Term Fasting in Menstrual Phases in Young Women: An Observational Study.” BMC Women's Health 15, no. 1: 67. 10.1186/s12905-015-0224-z. 26311347 PMC4551691

[psyp70017-bib-0085] Oostenveld, R. , P. Fries , E. Maris , and J.‐M. Schoffelen . 2011. “FieldTrip: Open Source Software for Advanced Analysis of MEG, EEG, and Invasive Electrophysiological Data.” Computational Intelligence and Neuroscience 2011: 156869. 10.1155/2011/156869. 21253357 PMC3021840

[psyp70017-bib-0086] Paleczny, B. , R. Seredyński , and B. Ponikowska . 2021. “Inspiratory‐ and Expiratory‐Gated Transcutaneous Vagus Nerve Stimulation Have Different Effects on Heart Rate in Healthy Subjects: Preliminary Results.” Clinical Autonomic Research 31, no. 2: 205–214. 10.1007/s10286-019-00604-0. 30941526 PMC8041682

[psyp70017-bib-0087] Pascual, F. T. 2015. “Vagus Nerve Stimulation and Late‐Onset Bradycardia and Asystole: Case Report.” Seizure 26: 5–6. 10.1016/j.seizure.2015.01.006. 25799894

[psyp70017-bib-0088] Patros, M. , D. G. S. Farmer , K. Moneghetti , et al. 2024. “First‐In‐Human Microelectrode Recordings From the Vagus Nerve During Clinical Vagus Nerve Stimulation.” Epilepsia Open 9, no. 6: 2522–2527. 10.1002/epi4.13083. 39465627 PMC11633718

[psyp70017-bib-0089] Patros, M. , D. G. S. Farmer , M. M. Ottaviani , et al. 2024. “Risk of Bradycardia and Asystole During Microelectrode Recordings From the Human Vagus Nerve.” Clinical Autonomic Research. 10.1007/s10286-024-01101-9. 39673646

[psyp70017-bib-0090] Patros, M. , M. M. Ottaviani , L. Wright , T. Dawood , and V. G. Macefield . 2022. “Quantification of Cardiac and Respiratory Modulation of Axonal Activity in the Human Vagus Nerve.” Journal of Physiology 600, no. 13: 3113–3126. 10.1113/JP282994. 35524982

[psyp70017-bib-0091] Penttilä, J. , A. Helminen , T. Jartti , et al. 2001. “Time Domain, Geometrical and Frequency Domain Analysis of Cardiac Vagal Outflow: Effects of Various Respiratory Patterns.” Clinical Physiology 21, no. 3: 365–376. 10.1046/j.1365-2281.2001.00337.x. 11380537

[psyp70017-bib-0092] Pervaz, I. , L. Thurn , C. Vezzani , L. Kaluza , A. Kühnel , and N. B. Kroemer . 2024. “Does Transcutaneous Vagus Nerve Stimulation Alter Pupil Dilation? A Living Bayesian Meta‐Analysis.” Neuroscience. 10.1101/2024.09.02.610851. 39884386

[psyp70017-bib-0093] Petzschner, F. H. , S. N. Garfinkel , M. P. Paulus , C. Koch , and S. S. Khalsa . 2021. “Computational Models of Interoception and Body Regulation.” Trends in Neurosciences 44, no. 1: 63–76. 10.1016/j.tins.2020.09.012. 33378658 PMC8109616

[psyp70017-bib-0094] Peuker, E. T. , and T. J. Filler . 2002. “The Nerve Supply of the Human Auricle.” Clinical Anatomy 15, no. 1: 35–37. 10.1002/ca.1089. 11835542

[psyp70017-bib-0095] Plassmann, H. , J. O'Doherty , and A. Rangel . 2007. “Orbitofrontal Cortex Encodes Willingness to Pay in Everyday Economic Transactions.” Journal of Neuroscience 27, no. 37: 9984–9988. 10.1523/JNEUROSCI.2131-07.2007. 17855612 PMC6672655

[psyp70017-bib-0096] Polanczyk, C. A. , L. E. P. Rohde , R. S. Moraes , E. L. Ferlin , C. Leite , and J. P. Ribeiro . 1998. “Sympathetic Nervous System Representation in Time and Frequency Domain Indices of Heart Rate Variability.” European Journal of Applied Physiology 79, no. 1: 69–73. 10.1007/s004210050475. 10052663

[psyp70017-bib-0097] Pomeranz, B. , R. J. Macaulay , M. A. Caudill , et al. 1985. “Assessment of Autonomic Function in Humans by Heart Rate Spectral Analysis.” American Journal of Physiology—Heart and Circulatory Physiology 248, no. 1: H151–H153. 10.1152/ajpheart.1985.248.1.H151. 3970172

[psyp70017-bib-0098] Prescott, S. L. , and S. D. Liberles . 2022. “Internal Senses of the Vagus Nerve.” Neuron 110, no. 4: 579–599. 10.1016/j.neuron.2021.12.020. 35051375 PMC8857038

[psyp70017-bib-0099] Qing, K. Y. , K. M. Wasilczuk , M. P. Ward , et al. 2018. “B Fibers Are the Best Predictors of Cardiac Activity During Vagus Nerve Stimulation: Qing, Vagal B Fiber Activation and Cardiac Effects.” Bioelectronic Medicine 4, no. 1: 5. 10.1186/s42234-018-0005-8. 32232081 PMC7098216

[psyp70017-bib-0100] Quintana, D. S. , and D. R. Williams . 2018. “Bayesian Alternatives for Common Null‐Hypothesis Significance Tests in Psychiatry: A Non‐technical Guide Using JASP.” BioMed Central Psychiatry 18, no. 1: 178. 10.1186/s12888-018-1761-4. 29879931 PMC5991426

[psyp70017-bib-0132] R Core Team . 2022. R: A Language and Environment for Statistical Computing. R Foundation for Statistical Computing.

[psyp70017-bib-0102] Redgrave, J. , D. Day , H. Leung , et al. 2018. “Safety and Tolerability of Transcutaneous Vagus Nerve Stimulation in Humans; a Systematic Review.” Brain Stimulation 11, no. 6: 1225–1238. 10.1016/j.brs.2018.08.010. 30217648

[psyp70017-bib-0103] Rong, P. , A. Liu , J. Zhang , et al. 2014. “Transcutaneous Vagus Nerve Stimulation for Refractory Epilepsy: A Randomized Controlled Trial.” Clinical Science. 10.1042/CS20130518. 24684603

[psyp70017-bib-0104] Ronkainen, E. , J. T. Korpelainen , E. Heikkinen , V. V. Myllylä , H. V. Huikuri , and J. I. T. Isojärvi . 2006. “Cardiac Autonomic Control in Patients With Refractory Epilepsy Before and During Vagus Nerve Stimulation Treatment: A One‐Year Follow‐Up Study.” Epilepsia 47, no. 3: 556–562. 10.1111/j.1528-1167.2006.00467.x. 16529621

[psyp70017-bib-0105] Scherrer, U. , and C. Sartori . 1997. “Insulin as a Vascular and Sympathoexcitatory Hormone: Implications for Blood Pressure Regulation, Insulin Sensitivity, and Cardiovascular Morbidity.” Circulation 96, no. 11: 4104–4113. 10.1161/01.CIR.96.11.4104. 9403636

[psyp70017-bib-0106] Sclocco, R. , R. G. Garcia , N. W. Kettner , et al. 2019. “The Influence of Respiration on Brainstem and Cardiovagal Response to Auricular Vagus Nerve Stimulation: A Multimodal Ultrahigh‐Field (7T) fMRI Study.” Brain Stimulation 12, no. 4: 911–921. 10.1016/j.brs.2019.02.003. 30803865 PMC6592731

[psyp70017-bib-0107] Shaffer, F. , and J. P. Ginsberg . 2017. “An Overview of Heart Rate Variability Metrics and Norms.” Frontiers in Public Health 5: 258. 10.3389/fpubh.2017.00258. 29034226 PMC5624990

[psyp70017-bib-0108] Shankar, R. , V. O. Olotu , N. Cole , H. Sullivan , and C. Jory . 2013. “Case Report: Vagal Nerve Stimulation and Late Onset Asystole.” Seizure 22, no. 4: 312–314. 10.1016/j.seizure.2012.12.011. 23290579

[psyp70017-bib-0109] Sharon, O. , F. Fahoum , and Y. Nir . 2021. “Transcutaneous Vagus Nerve Stimulation in Humans Induces Pupil Dilation and Attenuates Alpha Oscillations.” Journal of Neuroscience 41, no. 2: 320–330. 10.1523/JNEUROSCI.1361-20.2020. 33214317 PMC7810665

[psyp70017-bib-0110] Šinkovec, M. , R. Trobec , T. Kamenski , N. Jerman , and B. Meglič . 2023. “Hemodynamic Responses to Low‐Level Transcutaneous Auricular Nerve Stimulation in Young Volunteers.” IBRO Neuroscience Reports 14: 154–159. 10.1016/j.ibneur.2023.01.010. 36824666 PMC9941060

[psyp70017-bib-0111] Skora, L. , A. Marzecová , and G. Jocham . 2024. “Tonic and Phasic Transcutaneous Auricular Vagus Nerve Stimulation (taVNS) Both Evoke Rapid and Transient Pupil Dilation.” Brain Stimulation 17, no. 2: 233–244. 10.1016/j.brs.2024.02.013. 38423207

[psyp70017-bib-0112] Soeki, T. , K. Koshiba , T. Niki , et al. 2014. “Effect of Ghrelin on Autonomic Activity in Healthy Volunteers.” Peptides 62: 1–5. 10.1016/j.peptides.2014.09.015. 25265271

[psyp70017-bib-0113] Soer, R. , M. W. M. C. Six Dijkstra , H. J. Bieleman , F. G. J. Oosterveld , and N. H. M. Rijken . 2021. “Influence of Respiration Frequency on Heart Rate Variability Parameters: A Randomized Cross‐Sectional Study.” Journal of Back and Musculoskeletal Rehabilitation 34, no. 6: 1063–1068. 10.3233/BMR-200190. 34024811

[psyp70017-bib-0114] Szulczewski, M. T. 2022. “Transcutaneous Auricular Vagus Nerve Stimulation Combined With Slow Breathing: Speculations on Potential Applications and Technical Considerations.” Neuromodulation: Technology at the Neural Interface 25, no. 3: 380–394. 10.1111/ner.13458. 35396070

[psyp70017-bib-0115] Teckentrup, V. , and N. B. Kroemer . 2024. “Mechanisms for Survival: Vagal Control of Goal‐Directed Behavior.” Trends in Cognitive Sciences 28, no. 3: 237–251. 10.1016/j.tics.2023.11.001. 38036309

[psyp70017-bib-0116] Teckentrup, V. , M. Krylova , H. Jamalabadi , et al. 2021. “Brain Signaling Dynamics After Vagus Nerve Stimulation.” NeuroImage 245: 118679. 10.1016/j.neuroimage.2021.118679.

[psyp70017-bib-0117] Teckentrup, V. , S. Neubert , J. C. P. Santiago , M. Hallschmid , M. Walter , and N. B. Kroemer . 2020. “Non‐invasive Stimulation of Vagal Afferents Reduces Gastric Frequency.” Brain Stimulation 13, no. 2: 470–473. 10.1016/j.brs.2019.12.018. 31884186

[psyp70017-bib-0118] Thayer, J. F. , A. L. Hansen , E. Saus‐Rose , and B. H. Johnsen . 2009. “Heart Rate Variability, Prefrontal Neural Function, and Cognitive Performance: The Neurovisceral Integration Perspective on Self‐Regulation, Adaptation, and Health.” Annals of Behavioral Medicine 37, no. 2: 141–153. 10.1007/s12160-009-9101-z. 19424767

[psyp70017-bib-0119] Thayer, J. F. , and R. D. Lane . 2007. “The Role of Vagal Function in the Risk for Cardiovascular Disease and Mortality.” Biological Psychology 74, no. 2: 224–242. 10.1016/j.biopsycho.2005.11.013. 17182165

[psyp70017-bib-0120] Van Baak, M. A. 2008. “Meal‐Induced Activation of the Sympathetic Nervous System and Its Cardiovascular and Thermogenic Effects in Man.” Physiology & Behavior 94, no. 2: 178–186. 10.1016/j.physbeh.2007.12.020. 18281067

[psyp70017-bib-0121] Van Gent, P. , H. Farah , N. Van Nes , and B. Arem . 2019. “HeartPy: A Novel Heart Rate Algorithm for the Analysis of Noisy Signals.” Transportation Research Part F: Traffic Psychology and Behaviour 66: 368–378. 10.1016/j.trf.2019.09.015.

[psyp70017-bib-0122] Vaz, M. , A. Turner , B. Kingwell , et al. 1995. “Postprandial Sympatho‐Adrenal Activity: Its Relation to Metabolic and Cardiovascular Events and to Changes in Meal Frequency.” Clinical Science 89, no. 4: 349–357. 10.1042/cs0890349. 7493434

[psyp70017-bib-0123] Ventura‐Bort, C. , and M. Weymar . 2024. “Transcutaneous Auricular Vagus Nerve Stimulation Modulates the Processing of Interoceptive Prediction Error Signals and Their Role in Allostatic Regulation.” Human Brain Mapping 45, no. 3: e26613. 10.1002/hbm.26613. 38379451 PMC10879907

[psyp70017-bib-0124] Vest, A. N. , G. Da Poian , Q. Li , et al. 2018. “An Open Source Benchmarked Toolbox for Cardiovascular Waveform and Interval Analysis.” Physiological Measurement 39, no. 10: 105004. 10.1088/1361-6579/aae021. 30199376 PMC6442742

[psyp70017-bib-0125] Villani, V. , M. Tsakiris , and R. T. Azevedo . 2019. “Transcutaneous Vagus Nerve Stimulation Improves Interoceptive Accuracy.” Neuropsychologia 134: 107201. 10.1016/j.neuropsychologia.2019.107201. 31562863

[psyp70017-bib-0126] Vosseler, A. , D. Zhao , L. Fritsche , et al. 2020. “No Modulation of Postprandial Metabolism by Transcutaneous Auricular Vagus Nerve Stimulation: A Cross‐Over Study in 15 Healthy Men.” Scientific Reports 10, no. 1: 20466. 10.1038/s41598-020-77430-2. 33235256 PMC7686306

[psyp70017-bib-0131] Watson, D. , L. A. Clark , and A. Tellegen . 1988. “Development and Validation of Brief Measures of Positive and Negative Affect: The PANAS Scales.” Journal of Personality and Social Psychology 54, no. 6: 1063–1070. 10.1037/0022-3514.54.6.1063. 3397865

[psyp70017-bib-0127] Weise, D. , M. Adamidis , F. Pizzolato , J.‐J. Rumpf , C. Fricke , and J. Classen . 2015. “Assessment of Brainstem Function With Auricular Branch of Vagus Nerve Stimulation in Parkinson's Disease.” PLoS One 10, no. 4: e0120786. 10.1371/journal.pone.0120786. 25849807 PMC4388709

[psyp70017-bib-0128] Wolf, V. , A. Kühnel , V. Teckentrup , J. Koenig , and N. B. Kroemer . 2021. “Does Transcutaneous Auricular Vagus Nerve Stimulation Affect Vagally Mediated Heart Rate Variability? A Living and Interactive Bayesian Meta‐Analysis.” Psychophysiology 58, no. 11: e13933. 10.1111/psyp.13933. 34473846

[psyp70017-bib-0129] Yakunina, N. , S. S. Kim , and E.‐C. Nam . 2017. “Optimization of Transcutaneous Vagus Nerve Stimulation Using Functional MRI.” Neuromodulation: Technology at the Neural Interface 20, no. 3: 290–300. 10.1111/ner.12541. 27898202

[psyp70017-bib-0130] Yoshida, K. , K. Saku , K. Kamada , et al. 2018. “Electrical Vagal Nerve Stimulation Ameliorates Pulmonary Vascular Remodeling and Improves Survival in Rats With Severe Pulmonary Arterial Hypertension.” Journal of the American College of Cardiology: Basic to Translational Science 3, no. 5: 657–671. 10.1016/j.jacbts.2018.07.007. 30456337 PMC6234524

