# Supplementary material for: Non‐Invasive Auricular Vagus Nerve Stimulation Decreases Heart Rate Variability Independent of Caloric Load
Source: Psychophysiology. 2025 Feb 25;62(2):e70017. doi: 10.1111/psyp.70017 (PMC11862327; doi:10.1111/psyp.70017)
Supplement: Supplementary file 1 — Data S1.. [file PSYP-62-e70017-s001.docx]

# Supplementary Information

Non-invasive auricular vagus nerve stimulation decreases heart rate variability independent of caloric load

Kristin Kaduk^1,4^, Alessandro Petrella^1^, Sophie J. Müller^1^, Julian Koenig^2^, &

Nils B. Kroemer^1,3,4*^

^1^ Department of Psychiatry and Psychotherapy, Tübingen Center for Mental Health, University of Tübingen, Tübingen, Germany

^2^ Department of Child and Adolescent Psychiatry, Psychosomatics and Psychotherapy, University of Cologne, Faculty of Medicine and University Hospital Cologne, Cologne, Germany

^3^ Section of Medical Psychology, Department of Psychiatry and Psychotherapy, Faculty of Medicine, University of Bonn, Bonn, Germany

^4^ German Center for Mental Health (DZPG), partner site Tübingen

# Supplementary Material A: Table with taVNS studies of the introduction and their HRV indices

Table S1. All cited taVNS studies with their corresponding HRV indices

|  | **Measured variables** | **taVNS-induced Effects** |
| --- | --- | --- |
| Forte et al., 2022 | RMMSD, SDNN, LF-HRV, HF-HRV | Increase in SDNN, RMMSD, HF-HRV |
| Geng, Yang et al., 2022 | RMSSD, SDRR, HF-HRV, pRR50 | Increase in RMSSD |
| Geng, Liu, et al., 2022 | RMSSD, SDRR, HF-HRV, LF/HF ratio, PRR50 | Increase in RMSSD, PRR50, SDRR, HF-HRV |
| Bretherton et al. 2019 | RMSSD, HF-HRV, pRR50, SD1, BRS, | Increase in RMSSD, pRR50, SD1, BRS, HF-HRV |
| De Couck et al., 2017 | LF, LF/HF ratio and SDNN | Study1: Increase SDNN  Study2: Increased LF, LF/HF and SDNN in women |
| Gauthey et al., 2020 | RMSSD, SDRR, LF-HRV, HF-HRV, LF/HF ratio, HR | Increase in LF/HF ratio |
| Machetanz et al. 2021 | RMSSD, SDNN, pNN50, SD1, SD2 | Increase in SDNN, RMSSD, pNN50, SD1, SD2 |
| Altinkaya et al., 2023 | RMSSD | Decrease in RMSSD |
| Antonino et al., 2017 | HR, HF-HRV, LF-HRV, LF/HF ratio | Decrease HR & LF/HF ratio |
| Clancy et al., 2014 | LF-HRV, HF-HRV, LF/HF-HRV | Decrease in LF/HF ratio |
| Weise et al., 2015 | LF-HRV, HF-HRV, LF/HF-HRV | Decrease in LF/HF ratio |
| Borges et al., 2019 | RMSSD | No sig. effect |
| Burger et al., 2019 | RMSSD | No sig. effect |
| Šinkovec et al., 2023 | LF/HF-HRV | No sig. effect |
| Ventura-Bort & Weymer, 2024 | RMSSD | No sig. effect |
| Villani et al. 2019 | LF-HRV, HF-HRV, LF/HF-HRV, HR | No sig. effect |

# Supplementary Material B: The Milkshake recipe

The milkshake (basic recipe see Sun et al., 2015) contained 250g milk (3.5% fat, heat-treated; Gut & Günstig, Edeka, Germany), 50g cream (30% fat, heat-treated; Gut & Günstig, Edeka, Germany) and 50g syrup (Hershey's Syrup, The Hershey Company, USA). This corresponds to a nutrient composition of 9.4g protein (12%), 23.7g fat (31%), and 43.6g carbohydrates (57%). The milkshake has an energy of 400 kcal (1674.7 kJ) and a volume of 350 ml. To offer participants a milkshake they would like to consume, we offered three flavors (strawberry, chocolate, caramel) with comparable nutrient composition. The chosen flavor was used for all sessions throughout the experiment.

# Supplementary Material C: Stimulation intensity

Table S2. Mean and standard deviation of the applied stimulation intensities

| **Intensity in mA (mean, sd)** | **sham*** | **taVNS*** | ***P_Boot_*** |
| --- | --- | --- | --- |
| **Left** | 2.84 ± 1.09 | 2.40 ± 1.13 | *.024 ** |
| **Right** | 2.95 ± 1.03 | 2.24 ± 1.00 | *< .001 **** |
| ***P_Boot_*** | .457 | .284 |  |

*Note. ** We expected a difference between taVNS and sham in the stimulation intensity

because they were matched between taVNS and sham on the sensory level

# Supplementary Material D: Description for automatic artefact detection of the R peaks and R-R intervals

We evaluated whether the minimum distance between valid R peaks is at least 0.6 s (corresponding to a minimum HR of 36 bpm), and the minimum height of the peaks should be the median of the ECG energy profile (Lanata et al., 2015). Invalid peaks are identified by more than four times the median of the absolute difference between the amplitude of peaks. Invalid R-R intervals deviate by a factor of 0.6 from the mode of R-R intervals or by twice the mode of the R-R intervals. Finally, a Hampel filter, recommended as a moving window for nonlinear data cleaning (Ghaleb et al., 2018), was applied to search for deviations between consecutive intervals as an alternative measure of the distance from the median of its neighboring observations. All detected deviations and their consecutive R-R intervals were deleted from the signal. On average, 97.1% (1769s, range: 81% - 100%) of a 30 min block of ECG recordings was further analyzed to derive cardiovascular indices (except for one blocks with 956 s of ECG data). Overall, we carefully processed the ECG data and checked for artifacts to ensure the validity and accuracy of our results.

# Supplementary Material E: raw data of the HRV and HR


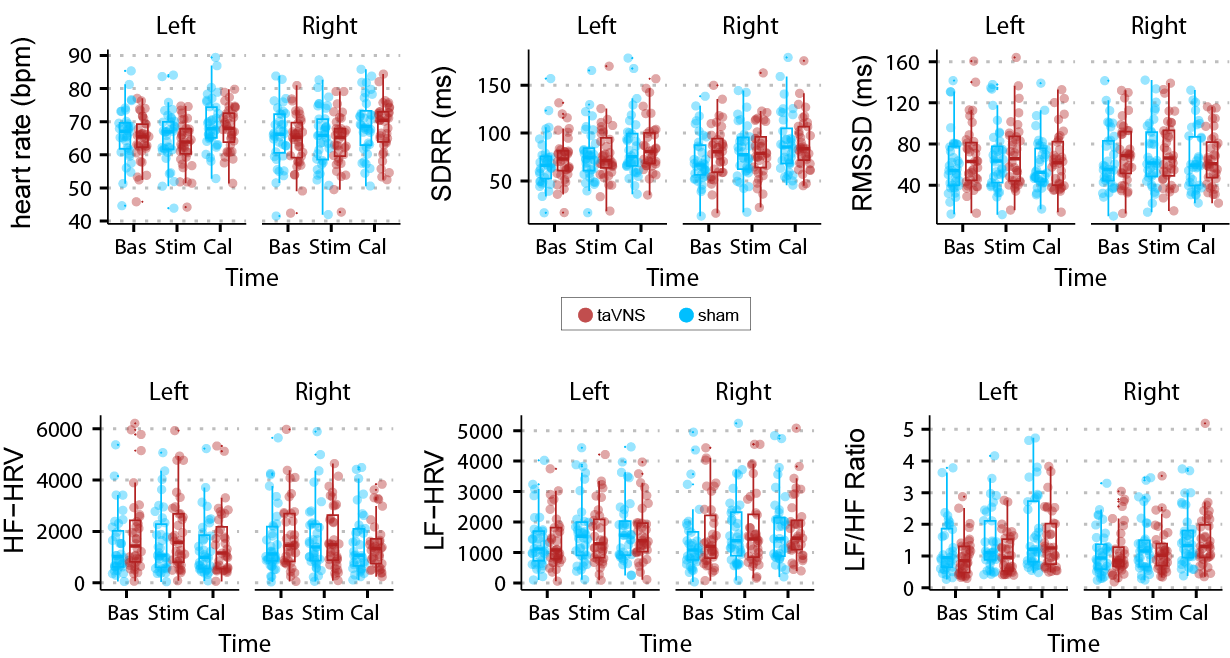


**Figure S1. taVNS decreases HRV, but not HR.** Displayed are the raw data for HR and the different HRV indices over time (baseline vs. stimulation vs. caloric load), respectively, for the two stimulation sides (left vs. right) and the two stimulation conditions (taVNS in red vs. sham in blue). The dots show the average score of each participant, and the boxplots show the interquartile range (IQR), where the line inside the box is the median, and the whiskers represent the minimum and maximum values within 1.5 times the IQR.

Table S3. Cardiovascular indices for the session-specific baseline: Mean and standard deviation

| DV | Side | taVNS | | sham | |
| --- | --- | --- | --- | --- | --- |
|  |  | mean | sd | mean | sd |
| HF (ms^2^) | Left | 1938.23 | 1635.14 | 1458.89 | 1198.37 |
| LF (ms^2^) |  | 1370.17 | 855.62 | 1346.55 | 871.85 |
| LF/HF ratio |  | 0.99 | 0.62 | 1.26 | 0.86 |
| RMSSD (ms) |  | 67.58 | 31.51 | 60.48 | 29.45 |
| SDRR (ms) |  | 73.46 | 23.81 | 69.14 | 26.10 |
| Heart rate (bpm) |  | 64.86 | 6.85 | 66.33 | 8.02 |
| HF (ms^2^) | Right | 1843.40 | 1342.77 | 1621.53 | 1295.38 |
| LF (ms^2^) |  | 1600.94 | 1152.18 | 1390.85 | 1099.53 |
| LF/HF ratio |  | 1.12 | 0.76 | 1.11 | 0.72 |
| RMSSD (ms) |  | 70.78 | 29.34 | 63.96 | 29.16 |
| SDRR (ms) |  | 80.48 | 27.59 | 72.82 | 26.66 |
| Heart rate (bpm) |  | 64.43 | 8.01 | 65.85 | 8.82 |

Table S4. Results of the multivariate analysis among all HRV indices for the baseline

|  | df | Approx. F | Trace | Num df | Den df | *p* |
| --- | --- | --- | --- | --- | --- | --- |
| Intercept | 1 | 561.784 | 0.943 | 4 | 137 | < .001 |
| Stim | 1 | 1.119 | 0.032 | 4 | 137 | .350 |
| Side | 1 | 0.706 | 0.020 | 4 | 137 | .589 |
| Side * Stim | 1 | 0.801 | 0.023 | 4 | 137 | .527 |

# Supplementary Material F: Analyses for the LF-HRV

Table S5. Results for the LF-HRV for the comparison taVNS vs. sham during stimulation phase after bootstrapping

| **Side** | **Time** | **mean** | **[CI_L_ , CI_U_]** |  | ***p*-value** |
| --- | --- | --- | --- | --- | --- |
| Both Sides | stimulation | -151.18 | -390.89, 44.79 |  | .175 |
|  | caloric load | -159.74 | -402.35, -45.59 |  | .162 |
| Left | stimulation | -122.27 | -391.88, 123.84 |  | .344 |
|  | caloric load | -97.30 | -343.52, 123.55 |  | .416 |
| Right | stimulation | -181.71 | -444.84, 52.13 |  | .137 |
|  | caloric load | -222.06 | -569.81, 83.88 |  | .167 |

*Note.* CI_L_ = 95% lower bound of confidence Interval; CI_U_ = 95% upper

bound of confidence Interval, *p* = p-value

# Supplementary Material G: HR and HRV over time


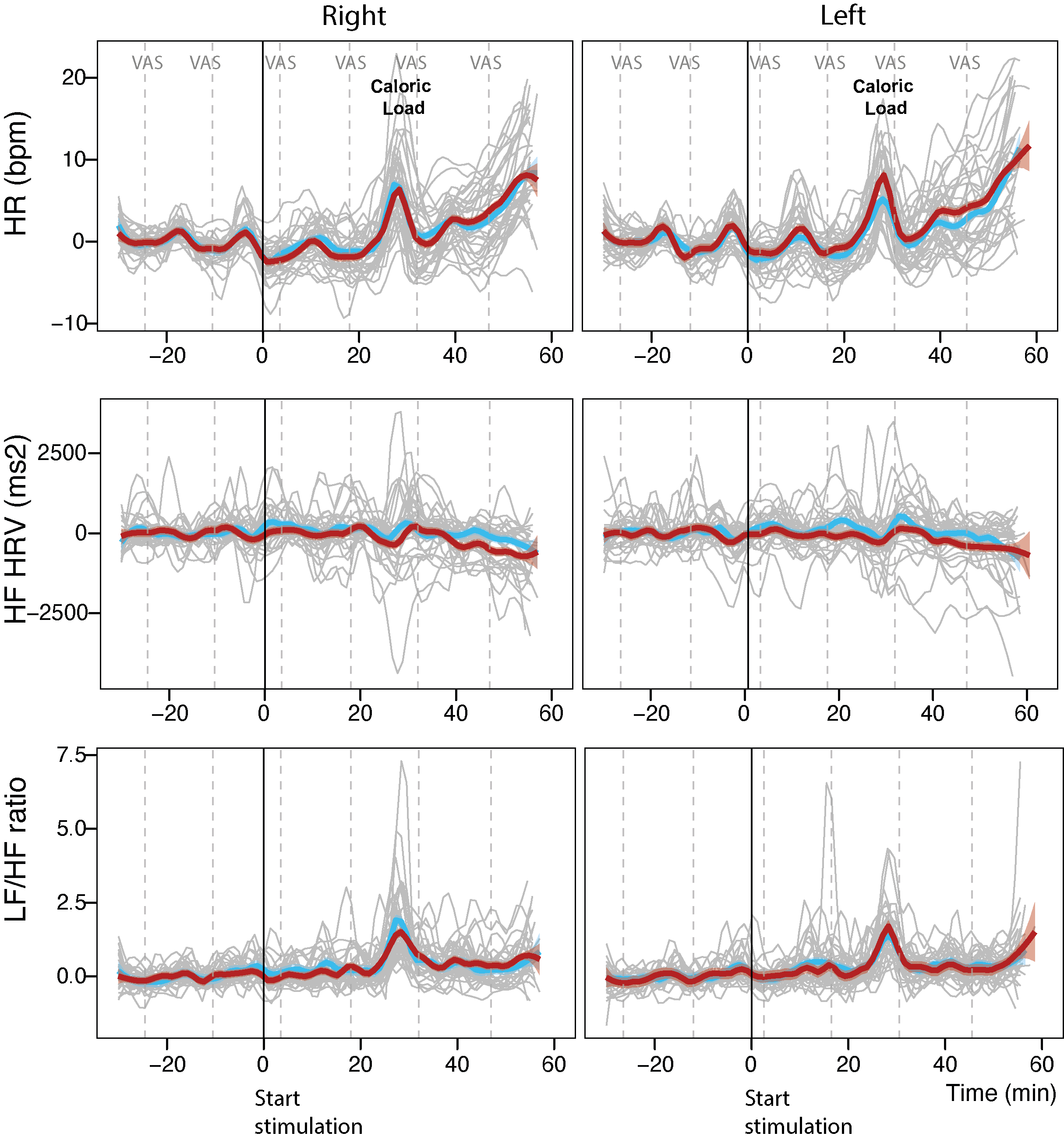


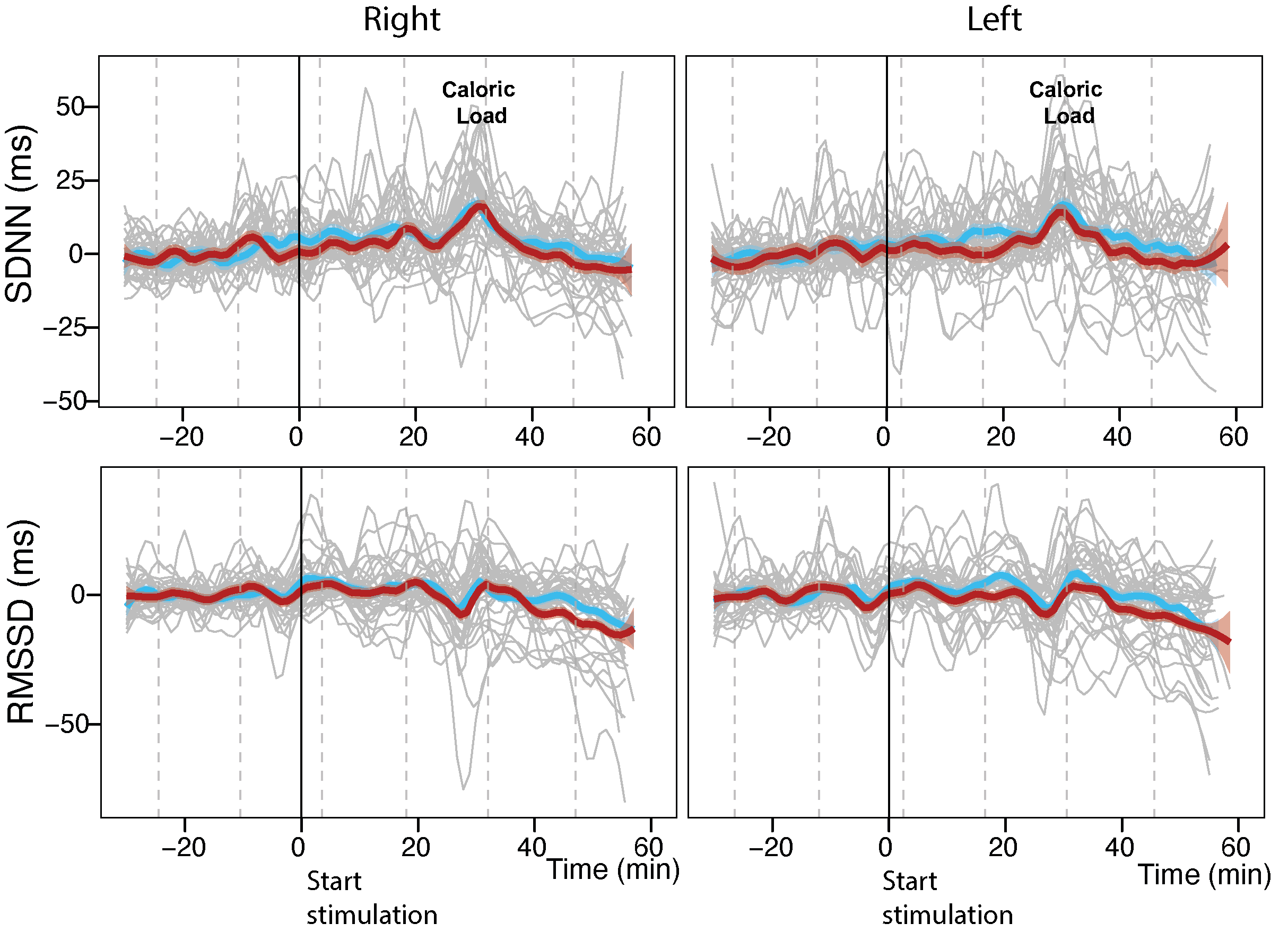


**Figure S2. taVNS decreases HRV but not HR over time.** Displayed are the relative changes in cardiac activity (HR, HF HRV, LF/HF ratio, SDRR, RMSSD) over time of the session, respectively, for the two stimulation sides (left vs. right) and the two stimulation conditions (taVNS in red vs. sham in blue). The data are aligned to the start of the stimulation. Individual participant data are shown in grey smoothed lines using loess regression (span = 0.1), while the overall trend for each stimulation group is depicted by thicker colored lines (span = 0.1). Vertical dashed grey lines represent approximate timings when participants filled out state ratings using a visual analog scale (VAS).

# Supplementary Material H: Table for the results in figure 4 C-E

Table S6. Correlations between right and left side for taVNS or sham

| **Variable** | **Stimulation** | **R** | ***p*-value** |  | **R** | ***p*-value** |
| --- | --- | --- | --- | --- | --- | --- |
|  | | Stimulation | |  | Caloric Load | |
| HR | Sham | **0.37** | **.027** |  | 0.18 | .280 |
|  | taVNS | **0.43** | **.008** |  | 0.32 | .058 |
| RMSSD | Sham | 0.28 | .102 |  | 0.11 | .517 |
|  | taVNS | **0.42** | **.011** |  | **0.48** | **.003** |
| SDRR | Sham | 0.25 | .149 |  | **0.56** | **< .001** |
|  | taVNS | **0.39** | **.019** |  | **0.58** | **< .001** |
| HF HRV | Sham | 0.09 | .611 |  | -0.07 | .702 |
|  | taVNS | **0.43** | **.009** |  | **0.58** | **< .001** |
| LF/HF ratio | Sham | 0.32 | .053 |  | **0.46** | **.005** |
|  | taVNS | **0.49** | **.002** |  | **0.47** | **.004** |

*Note.* R = Pearson-Correlation-Coefficient

# Supplementary Material I: Bayes factors for the side-specific effects

Table S7. Bayes factors for the side-specific effects

| **Variable** | HR | RMSSD | SDRR | HF HRV | LF/HF ratio |
| --- | --- | --- | --- | --- | --- |
| **BF_10_** | 0.560 | 0.236 | 0.424 | 0.184 | 0.235 |

*Note.* BF_10_ = Bayes Factor, interpretation according to

Jeffreys (1939) with BF_10_ < 1 evidence favours null hypothesis

# Supplementary Material J: Comparison between both phases

Table S8. Results of difference in taVNS-induced effects between both phases (Stimulation vs. Caloric load)

| **Variable** | **Contrast** | **mean** | **[CI_L_ , CI_U_]** |  | ***p*-value** |
| --- | --- | --- | --- | --- | --- |
| RMSSD | Stim vs.Cal | -1.04 | -4.26, -2.22 |  | .531 |
| SDRR | Stim vs.Cal | -0.19 | -3.63, -3.07 |  | .922 |
| HF HRV | Stim vs.Cal | -95.88 | -237.32, 41.26 |  | .171 |
| LF/HF ratio | Stim vs.Cal | 0.10 | -0.04, 0.23 |  | .152 |
| Heart rate | Stim vs.Cal | 0.60 | -0.03, 1.22 |  | .063 |

*Note.* CI_L_ = 95% lower bound of confidence Interval; CI_U_ = 95% upper

bound of confidence Interval, *p* = p-value
